# Genomic and Phenotypic Biology of Novel Strains of *Dickeya zeae* Isolated From Pineapple and Taro in Hawaii: Insights Into Genome Plasticity, Pathogenicity, and Virulence Determinants

**DOI:** 10.3389/fpls.2021.663851

**Published:** 2021-08-11

**Authors:** Gamze Boluk, Dario Arizala, Shefali Dobhal, Jingxin Zhang, John Hu, Anne M. Alvarez, Mohammad Arif

**Affiliations:** ^1^Department of Plant and Environmental Protection Sciences, University of Hawai’i at Mānoa, Honolulu, HI, United States; ^2^Institute of Plant Protection, Guangdong Academy of Agricultural Sciences, Guangzhou, China

**Keywords:** *Dickeya zeae*, comparative genomics, pectinolytic bacteria, phylogenomics, pathogenicity determinants and virulence factors, taro (*Colocasia esculenta*), pineapple

## Abstract

*Dickeya zeae*, a bacterial plant pathogen of the family Pectobacteriaceae, is responsible for a wide range of diseases on potato, maize, rice, banana, pineapple, taro, and ornamentals and significantly reduces crop production. *D. zeae* causes the soft rot of taro (*Colocasia esculenta*) and the heart rot of pineapple (*Ananas comosus*). In this study, we used Pacific Biosciences single-molecule real-time (SMRT) sequencing to sequence two high-quality complete genomes of novel strains of *D. zeae*: PL65 (size: 4.74997 MB; depth: 701x; GC: 53.6%) and A5410 (size: 4.7792 MB; depth: 558x; GC: 53.5%) isolated from economically important Hawaiian crops, taro, and pineapple, respectively. Additional complete genomes of *D. zeae* representing three additional hosts (philodendron, rice, and banana) and other species used for a taxonomic comparison were retrieved from the NCBI GenBank genome database. Genomic analyses indicated the truncated type III and IV secretion systems (T3SS and T4SS) in the taro strain, which only harbored one and two genes of T3SS and T4SS, respectively, and showed high heterogeneity in the type VI secretion system (T6SS). Unlike strain EC1, which was isolated from rice and recently reclassified as *D. oryzae*, neither the genome PL65 nor A5410 harbors the zeamine biosynthesis gene cluster, which plays a key role in virulence of other *Dickeya* species. The percentages of average nucleotide identity (ANI) and digital DNA–DNA hybridization (dDDH) between the two genomes were 94.47 and 57.00, respectively. In this study, we compared the major virulence factors [plant cell wall-degrading extracellular enzymes and protease (Prt)] produced by *D. zeae* strains and evaluated the virulence on taro corms and pineapple leaves. Both strains produced Prts, pectate lyases (Pels), and cellulases but no significant quantitative differences were observed (*p* > 0.05) between the strains. All the strains produced symptoms on taro corms and pineapple leaves, but the strain PL65 produced symptoms more rapidly than others. Our study highlights the genetic constituents of pathogenicity determinants and genomic heterogeneity that will help to understand the virulence mechanisms and aggressiveness of this plant pathogen.

## Introduction

*Dickeya* and *Pectobacterium* are Gram-negative, rod-shaped bacteria, which belongs to the family Pectobacteriaceae (order Enterobacteriales), and are devastating phytopathogens (Adeolu et al., 2016). *Dickeya* species have been listed among the top 10 most important bacterial phytopathogens due to their high economic consequences (Mansfield et al., 2012). *Dickeya* currently encompasses 12 recognized species, namely *D. chrysanthemi*, *D. paradisiaca*, *D. zeae*, *D. dianthicola*, *D. dadantii* (Samson et al., 2005), *D. solani* (van der Wolf et al., 2014), *D. aquatica* (Parkinson et al., 2014), *D. fangzhongdai* (Tian et al., 2016), *D. lacustris* (Hugouvieux-Cotte-Pattat et al., 2019), *D. undicola* (Oulghazi et al., 2019), *D. poaceiphila* (Hugouvieux-Cotte-Pattat et al., 2020), and *D. oryzae*—recently separated from *D. zeae* (Wang et al., 2020). *Dickeya dadantii* has two subspecies, *D. dadantii* subsp. *dadantii* and *D. dadantii* subsp. *dieffenbachiae* (Brady et al., 2012). The *D. zeae* strains were isolated from a wide and diverse range of hosts such as pineapple, potato, maize, rice, banana, hyacinth, clivia, *Brachiaria*, chrysanthemum, and philodendron (Sinha and Prasad, 1977; Samson et al., 2005; Sławiak et al., 2009; Toth et al., 2011; Li et al., 2012; Bertani et al., 2013; Pritchard et al., 2013; Zhang et al., 2014; Hu et al., 2018). Among the *Dickeya* species, *D. solani*, *D. dadantii*, and *D. zeae* often cause serious economic losses, especially on potato, rice, pineapple, and banana (Hussain et al., 2008; Sławiak et al., 2009; Lin et al., 2010; Toth et al., 2011; Zhou J. et al., 2011; Marrero et al., 2013; Zhang et al., 2014).

*Dickeya zeae* is diverse and affects several hosts, including maize. Bacterial strains associated with pineapple heart rot disease in Hawaii were identified as the strains of *D. zeae* as the closest match, although distinguishing features appeared to warrant the description as a new species (Marrero et al., 2013). Multilocus sequencing typing (*gapA*, *purA*, *gyrB*, *atpD*, and *dnaA*) analysis of pineapple strains showed high similarity with *D. oryzae* (Boluk and Arif, unpublished information), and recently, the rice pathogen, *D. oryzae* was separated from *D. zeae* (Wang et al., 2020). Thus, further phylogenetic analysis is warranted.

Phytopathogens can reside on the surfaces and/or within the intercellular spaces of plant leaves, without exhibiting symptoms (Pérombelon, 1992). When the optimal conditions of temperature, humidity, and other factors occur, bacteria proliferate and produce plant cell-wall-degrading enzymes (PCWDEs) leading to disease development (Hugouvieux-Cotte-Pattat et al., 1996; Pérombelon, 2002). *Dickeya* species cause soft rot *via* a coordinated production of various secreted enzymes, mainly PCWDEs, including pectinases, cellulases, and proteases (Prts) (Hugouvieux-Cotte-Pattat et al., 1996), which constitute the primary and most essential virulence determinants (Toth et al., 2006; Davidsson et al., 2013). These PCWDEs play a significant role in bacterial pathogenesis by macerating host plant tissues and enabling host colonization and disease development (Collmer and Keen, 1986; Charkowski et al., 2012, Charkowski et al., 2014; Davidsson et al., 2013). The plant cell wall is a complex of polymers [cellulose (Cel), hemicellulose, pectin, and structural glycoproteins] (Pauly and Keegstra, 2016), and among these polymers, pectin is the most complex and includes both polygalacturonan and ramified regions [rhamnogalacturonan I (RGI) and RGII, respectively] (Caffall and Mohnen, 2009). RGI contains a rhamnogalacturonan backbone and various lateral chains such as galactan, arabinan, and galacturonan (Caffall and Mohnen, 2009). RGII contains a short galacturonan backbone, carrying four side chains (O’Neill et al., 2004). Methyl esterification and acetylation groups of pectin are removed by pectin methylesterases (Pem) and pectin acetylesterase (Pae) (Hugouvieux-Cotte-Pattat et al., 2014).

**FIGURE 1:**
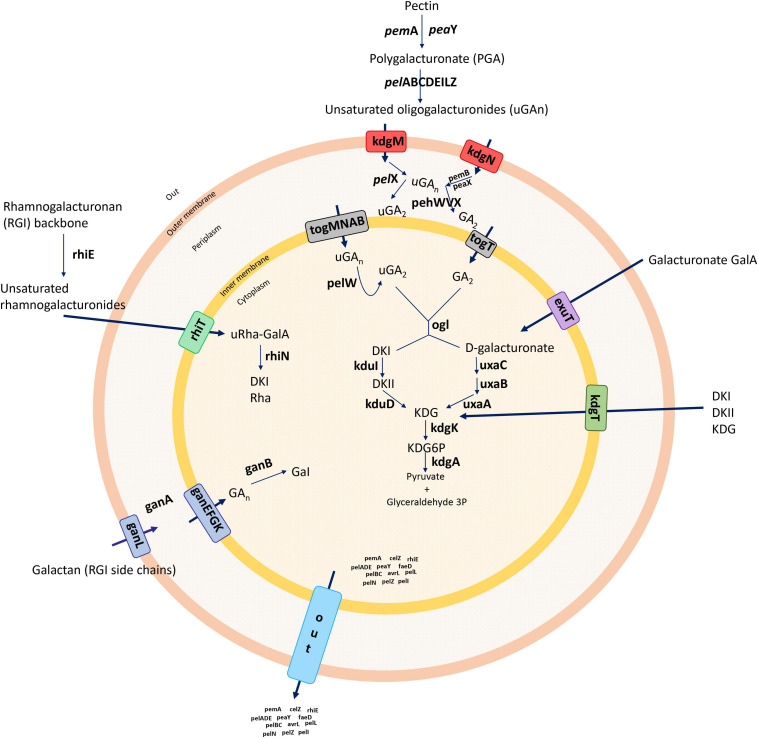
Summary of the general pectin degradation pathway in soft rot bacteria.

Unsaturated oligogalacturonates enter the periplasm using the transporters KdgM and KdgN (Charkowski et al., 2012). Upon entry into the periplasm, the oligomers are further cleaved by polygalacturonases (Pehs) (Hugouvieux-Cotte-Pattat et al., 2014). The small oligomers enter the cytoplasm using the transporters TogT and TogMNAB after conversion into D-galacturonate and 4-deoxy-L-threo-5-hexosulose uronic acid by oligogalacturonate lyase (Ogl) (Hugouvieux-Cotte-Pattat et al., 2014). The oligomers are catabolized into 3-phosphoglyceraldehyde by the enzymes KduID, KdgK, and UxaABC in the cytoplasm (Hugouvieux-Cotte-Pattat, 2016). Additionally, D-galacturonate and 4-deoxy-L-threo-5-hexosulose uronic acid can enter the cytoplasm directly using the transporters ExuT and KdgT, respectively (Hugouvieux-Cotte-Pattat, 2016). The rhamnogalacturonate lyase (*rhi*) genes are involved in the degradation of the RGI pectin-ramified regions (Hugouvieux-Cotte-Pattat et al., 2014), and the resulting RGI is cleaved by RhiE, which leads the oligomers to enter the cytoplasm through the transporter RhiT (Hugouvieux-Cotte-Pattat et al., 2014). In the cytoplasm, the enzyme RhiN cleaves the unsaturated galacturonate. The periplasmic endo-galactanase (Gan) gene cluster is responsible for the enzymes that destroy galactan chains—the galactans enter the periplasm by the transporter GanL (Hugouvieux-Cotte-Pattat et al., 2014). GanA generates short oligomers, which use the GanFGK transport system to cross an inner membrane (Hugouvieux-Cotte-Pattat et al., 2014), and finally, GanB cleaves oligogalactan to galactose (Figure 1; Hugouvieux-Cotte-Pattat and Reverchon, 2001; Hugouvieux-Cotte-Pattat et al., 2014; Hugouvieux-Cotte-Pattat, 2016). In pectinolytic bacteria, Prts play a significant role in virulence mechanisms, and unlike PCWDE, these enzymes are associated with the type I secretion system (T1SS) (Toth et al., 2006; Charkowski et al., 2012). The secreted exoenzymes from the type II secretion system (T2SS), known as the *out* operon, are secreted from the cytoplasm to an extracellular space (Hugouvieux-Cotte-Pattat et al., 1996, 2014; Toth et al., 2006). Type secretion systems (T1SS–T6SS) release and modulate the transport of most previously described virulence components (Hugouvieux-Cotte-Pattat et al., 1996; Toth et al., 2006). Therefore, protein secretion systems are considered as the core set of players that regulate the mechanism of pathogenesis in *Dickeya* (Charkowski et al., 2012). The T3SS, which forms an injection machinery needed to infect plants and transport virulence proteins into the cytoplasm, is one of the major components of pathogenesis and in the hypersensitivity reaction (Hrp) (Alfano and Collmer, 2004; Yap et al., 2005; Charkowski et al., 2012). The T4SS referred to as a conjugation system involved in bacterial DNA transfer delivers effector proteins (virulence factors) directly to the host during infection *via* a cell contact-dependent way (Trokter and Waksman, 2018). The T6SS, possibly important for bacterial pathogenicity and host adaptation, has been associated with biofilm formation and bacterial survival (Masum et al., 2017). In addition to protein secretion systems, bacterial pathogens form biofilms with complicated matrices including bacterial secretions that bind to plant surfaces and enhance the capacity of bacteria to infect the host (O’Toole and Kolter, 1998). Biofilm development and surface attachment are accelerated by functional flagella (O’Toole and Kolter, 1998). Flagella and chemotaxis are important for the establishment of successful infections (Jahn et al., 2008). Furthermore, type IV pili are responsible for surface-associated motility (twitching motility), which allows bacteria to anchor, retract, and push forward, in advancing the cells (O’Toole and Kolter, 1998). Cell motility, secretion, and vesicular transport are generally associated with flagellar proteins, whereas signal transduction occurs with chemotaxis proteins (Jahn et al., 2008). The pilus structures are linked to main virulence functions, namely adhesion, bacterial conjugation, surface motility, and the interactions between bacteria and host cells (Craig et al., 2004; Maier and Wong, 2015). Another important feature of biofilm development is the ability of bacteria to biosynthesize polysaccharides (Watnick et al., 2001). Exopolysaccharide synthesis plays a vital role in forming a three-dimensional architecture of biofilms (Watnick et al., 2001). The polysaccharides support multiple biological processes, such as a bacterial attachment to the host, colonization, virulence, and the protection from plant toxins and extreme environmental conditions (Toth et al., 2006).

In this study, we first aim to understand the genomic constituents of diverse *D. zeae* strains and arsenals involved in pathogenicity and host adaptation through comparative genomic analyses; and secondly, to understand the genomic and phenotypic biology of the two novel strains, 5410 and PL65, isolated in Hawaii from pineapple and taro, respectively.

## Materials and Methods

### Bacterial Strains and Genomic DNA Extraction

Two novel *Dickeya* strains (A5410 and PL65) are representative of multiple bacterial strains isolated from pineapple and taro grown in Hawaii. Pineapples, planted with suckers imported from Costa Rica and the Philippines, exhibited heart rot symptoms in Hawaii (Sueno et al., 2014). Strain A5410 was isolated in August 2007 from pineapple leaves (*Ananas comosus*) showing symptoms of pineapple heart rot. Strain PL65 was isolated in 2018 from a taro corm (*Colocasia esculenta*), showing soft rot symptoms. These and similar strains are maintained in the Pacific Bacterial Collection, the University of Hawai’i at Mānoa, Honolulu, HI, United States.

Bacteria were streaked on dextrose peptone agar (DPA: peptone 10 g/l, dextrose 5 g/l, and agar 17 g/l) (modified from Norman and Alvarez, 1989) and incubated at 28°C for 24 h. A single colony was streaked onto DPA and incubated at 28°C for 24 h.

A half loopful of overnight grown bacterial culture was used to extract the genomic DNA using the QIAGEN Genomic-tip 100/G (Qiagen, Valencia, CA, United States) according to the instructions of the manufacturer. Quantification and quality control of the DNA were performed using a Nanodrop spectrophotometer, and a Qubit 4 fluorometer (Thermo Fisher Scientific, Life Technologies, Carlsbad, CA, United States).

### Whole-Genome Sequencing and Annotation

Whole-genome sequencing of both strains was performed at the Washington State University facility using a PacBio RS II (Pacific Biosciences of California, Inc., Menlo Park, CA, United States) with a single-molecule real-time (SMRT). The libraries were prepared with a 20 kb insert size and sequenced using C4 sequencing chemistry and P6 polymerase. The sequencing reads were trimmed based on quality and length to generate highly accurate long reads and further assembled using the Hierarchical Genome Assembly Process HGAP v4 (Pacific Biosciences, SMRT Analysis Software v2.3.0). The assembled genomes were annotated using the three different pipelines: the NCBI prokaryotic genome annotation pipeline (PGAP) (Tatusova et al., 2016), the Integrated Microbial Genomes pipeline version 4.10.5 from the Joint Genome Institute (IMG-JGI; Huntemann et al., 2015), and the Rapid Annotation System Technology (RAST) server (Brettin et al., 2015). Both genomes, A5410 and PL65, were submitted to the NCBI GenBank genome database under the accession numbers CP040816 and CP040817, respectively.

### Comparative Genomics and Phylogenomic Analyses of *Dickeya* Species

Thirteen genomes, including *Dickeya aquatica* 174/2, *D. chrysanthemi* Ech1591, *D. dadantii* 3937, *D. dianthicola* ME23, *D. fangzhongdai* PA1, *D. lacustris* S29, *D. paradisiaca* Ech703, *D. solani* IPO 2222, *D. undicola* FVG10-MFV-A16, *D. zeae* EC1, Ech586, and MS2, and *Pectobacterium atrosepticum* 36A, were retrieved from the NCBI GenBank genome database on February 2, 2020 (Table 1). A pairwise comparison of A5410 and PL65 genomes with the 13 genomes was performed using the average nucleotide identity (ANI) based on the Nucleotide MUMmer algorithm (ANIm) in Jspecies Web Server (Richter et al., 2016). The digital DNA–DNA hybridization (dDDH) was calculated using the genome-to-genome distance calculator (GGDC)^1^ version 2.1 with the recommended formula two and BLAST+ alignment criteria. The ANI and dDDH data were compiled in a single matrix and visualized as a color-coded heatmap using DISPLAYR^2^. The cut-off values of 95–96% (Goris et al., 2007; Richter and Rosselló-Móra, 2009; Kim et al., 2014; Chun et al., 2018; Wang et al., 2020) and 70% (Wayne et al., 1987; Goris et al., 2007) were assigned as a species-delineation framework for ANI and dDDH, respectively. The ANI phylogenetic tree was generated for the strains of various *Dickeya* species based on the whole-genome alignment using the neighbor-joining method. The Jukes–Cantor model was used for analysis, and the tree was built based on 1,000 bootstraps—CLC Workbench 20 was used for analyses.

**TABLE 1:** Selected genomes of *Dickeya* and *Pectobacterium* used for comparative and phylogenomic analyses.

Species	Strain name	NCBI accession number	Location	Host/Source	Isolation year	Replicons (Assembly level)	Genome size (Mb)	GC%
*Dickeya zeae*	EC1*	NZ_CP006929	China	*Oryza sativa*	–	1 contig	4.53	53.4
*Dickeya* sp.	A5410	NZ_CP040816	United States	*Ananas comosus*	2007	1 contig	4.78	53.5
*Dickeya* sp.	PL65	NZ_CP040817	United States	*Colocasia esculenta*	2018	1 contig	4.75	53.6
*D. zeae*	Ech586	NC_013592	United States Florida	*Philodendron* Schott	–	1 contig	4.82	53.6
*D. zeae*	MS2	NZ_CP025799	China	*Musa* sp.	2014	1 contig	4.74	53.4
*Dickeya aquatica*	174/2^*T*^	NZ_LT615367	United Kingdom	River water	2012	1 contig	4.5	53.6
*Dickeya chrysanthemi*	Ech1591	NC_012912	–	*Zea mays*	–	1 contig	4.81	54.5
*Dickeya dadantii*	3937	NC_014500	France	*Saintpaulia ionantha*	1977	1 contig	4.92	56.3
*Dickeya dianthicola*	ME23	NZ_CP031560	United States Maine	*Solanum tuberosum*	2016	1 contig	4.91	55.7
*Dickeya fangzhongdai*	PA1	NZ_CP020872	China	*Phalaenopsis* sp.	2011	1 contig	4.98	56.9
*Dickeya lacustris*	S29^*T*^	NZ_QNUT01	France	*River water*	2017	118 contigs	4.31	53.1
*Dickeya paradisiaca*	Ech703	NC_012880	Australia	*S. tuberosum*	–	1 contig	4.68	55
*Dickeya solani*	IPO 2222^*T*^	NZ_CP015137	Netherlands	*S. tuberosum*	2007	1 contig	4.92	56.2
*Dickeya undicola*	FVG10-MFV-A16	NZ_RJLS00	France	Fresh water	2017	202 contigs	4.54	54.5
*Pectobacterium atrosepticum*	36A	NZ_CP024956	Belarus	*S. tuberosum*	1978	1 contig	4.97	51.1

*Type strains are marked with “^*T*^” after the strain name.*

The data that are not available are marked with “–”.

*EC1 was *Dickeya zeae*, but recently, this strain has been reclassified as *D. oryzae.*

Blast matrix, codon usage, amino acid usage, and pan-core analyses across the *Dickeya* strains were analyzed using the CMG-biotools pipeline (Vesth et al., 2013). The percentage of shared proteins among the *Dickeya* strains was computed based on 50/50 Basic Local Alignment Search Tool (BLAST) analysis (50% identity match and 50% length identity). The generated BLAST matrix plot was visualized as a color scale heatmap showing the numerical homology percentages across all compared proteomes. Besides, a clustering analysis according to codon and amino acid usage data was determined for the genomes that displayed DDH, ANI, ANIm, and TETRA values below the cut-off parameter for species delineation compared with the reference strain of affiliated *Dickeya* species. The codon and amino acid usage were calculated using BioPerl modules (Stajich et al., 2002) and visualized as heatmaps using R as implemented in the CMG-biotools (Vesth et al., 2013).

The pan-core genome plot, tree analyses, and predicted proteome comparisons were performed with the genomes of 14 *Dickeya* species using CMG-biotools (Vesth et al., 2013). Pairwise pan- and core-genomes were calculated for all genome combinations as mentioned above using the BLAST algorithm (Altschul et al., 1990) with 50% cut-off values for either query cover or identity percentage parameters. Core- and pan-genome plots were visualized in the pan-core plot program using CMG-biotools (Vesth et al., 2013).

The phylogenetic relationship was performed based on multilocus sequence analyses using 86 virulence-related genes (most are involved in cell wall-degrading enzyme gene clusters) (Supplementary Table 1). The corresponding gene sequences of 14 *Dickeya* species and 1 *Pectobacterium* species (used as an out-group) were retrieved from the NCBI GenBank genome database (Table 1). Eight-six concatenated gene sequences were aligned using the progressiveMauve plugin in Geneious Prime v 2020.0.4. The concatenated alignment data were used to generate the Neighbor-Joining phylogenetic tree using CLC Genomics Workbench 20 (Qiagen, Valencia, CA, United States).

### Genome Comparisons of *D. zeae* Species Complex

The complete genomes of both novel *Dickeya* sp. strains (A5410 and PL64) were compared with the other three complete genomes of *D. zeae* (EC1, Ech586, and MS2). Recently, *D. zeae* strain EC1 was reclassified as *D. oryzae* (Wang et al., 2020), a novel species within the genus *Dickeya*. However, we included the genome of EC1 in our analyses due to its close relationship to *D. zeae* strains. Complete genomes of EC1, Ech586, and MS2 were retrieved from the NCBI genome database. The basic genomic profile features of the two novel strains were taken from the NCBI GenBank database and the Bioinformatic Resource Center Proteome Comparison tool of Pathosystems Resource Integration Center (PATRIC) Web server (Wattam et al., 2014, 2017; Supplementary Table 2). Additionally, the genome atlases were constructed to illustrate different structural components present in the DNA sequences such as a guanine-cytosine (GC) skew, stacking energy, an intrinsic curvature, a position preference, global direct, and indirect repeats. The previous parameters were visualized and drawn as a circle plot using the GeneWiz program (Hallin et al., 2009), which were outputted in the workbench CMG-biotools by using the script atlas_createConfig (Vesth et al., 2013). Genomic islands (Gis) were predicted using the IslandViewer 4 webserver (Bertelli et al., 2017) for both the new strains. IslandPath-DIMOB (Bertelli and Brinkman, 2018), SIGI-HMM, IslandPick (Langille et al., 2008), and Islander (Hudson et al., 2015) were used to generate an interactive visualization of Gis.

Multi-genome alignment of five genomes was conducted using the progressiveMauve 2.3.1 (Darling et al., 2010). A set of common (core genome) and unique genes within the *Dickeya* genus were identified using an all-against-all comparison determined with an OrthoMCL pipeline using the BLASTP algorithm all-against-all genomes comparison included in this study (Li et al., 2003). The orthologous gene clustering analyses were implemented with default settings. OrthoMCL clustering analyses were performed with the following parameters: value of *p* cut-off = 1 × 10^–5^; identity cut-off = 90%; percent match cut-off = 80 (Li et al., 2003).

The clusters involved in various virulence and pathogenicity functions (such as the PCWDE), types of secretion systems (I–VI), the synthesis of polysaccharides [enterobacterial common antigen (ECA), capsular polysaccharide (CPS), lipopolysaccharides (LPSs), exopolysaccharides, and O-antigen], bacterial attachment operons (type IV pili), and flagella and chemotaxis were screened and compared among the *D. zeae* complex using the PATRIC web server (Wattam et al., 2017). The syntenic and different rearrangements between the main pathogenicity genomic clusters were visualized as linear arrows generated using Easyfig v2.2.3 (Sullivan et al., 2011). The secondary metabolic biosynthetic-related gene clusters were predicted using antiSMASH 4.0 (Blin et al., 2017). The Clustered Regularly Interspaced Short Palindromic Repeats (CRISPRs) arrays and the types of CRISPR-associated proteins (Cas) systems were predicted using CRISPRCasFinder (Couvin et al., 2018). The prophage identification tool PHAge Search Tool Enhanced Release (PHASTER) was used to search for the regions containing prophage-like elements in bacterial genomes (Edmonton, AB, Canada)^3^ (Zhou Y. et al., 2011; Arndt et al., 2016, Arndt et al., 2019).

### Phenotypic Comparisons of Novel Strains

A type strain of *D. zeae*, NCPPB 2538 (=A5422; CFBP 2052) and the novel strains A5410 and PL65 were phenotypically characterized for the production of extracellular enzymes, swimming, and swarming, polysaccharide synthesis, biofilm formation, and pathogenicity following the different protocols described below. Initially, the growth curve was evaluated for all three strains.

#### Pel, Cel, and Prt Enzyme Activity Assays

The protocol reported by Chatterjee et al. (1995) was followed for the enzyme activity assays using three mediums as follows: (1) Pel assay medium (per liter):10 g polygalacturonic acid (PGA), 10 g yeast extract, 0.38 μmol CaCl_2_, 100 mmol Tris–HCl, pH 8.5, 8 g agarose, and 2 g sodium azide; (2) cellulose (Cel) assay medium:1 g carboxymethyl Cel and 25 mM sodium phosphate, pH 7.0, 8 g agarose, and 2 g sodium azide; (3) Prt assay medium: 30 g gelatin, 4 g nutrient, 8 g agar, and 2 g sodium azide. The media were poured and allowed to solidify and 3-mm-diameter wells were punched into the agar and sealed at the bottom with molten agarose. A bacterial suspension (50 μl of overnight culture; OD_600_∼1.2) was applied to each well, and plates were incubated at 28°C. After 10 h, Pel assay plates were flooded with 4 N HCl, and Cel assay plates were flooded with 2% Congo red solution for 10 min and washed for 5 min with 5 M NaCl. Haloes around the wells in Prt plates within 24 h indicated that Prt activity diameter of a clear halo around the colonies was measured. Each treatment was carried out three times, and all assays were repeated three times.

#### Motility Assay

Swimming and swarming motility assays were performed in a semi-solid medium as described by Chen et al. (2016). The swimming medium per liter contained 10 g tryptone, 5 g NaCl, and 3 g agar supplemented with 0.05% (w/v) tetrazolium chloride while the swarming medium per liter contained 10 g tryptone, 5 g NaCl, and 4 g agar supplemented with 0.05% (w/v) tetrazolium chloride A single, pure bacterial colony was inoculated at the center of each plate using a toothpick. Plates were incubated at 28°C for 24 h, and the diameter of the bacterial growth was measured. Each treatment was carried out three times, and the assays were repeated three times.

#### Exopolysaccharide Production Assay

The exopolysaccharide production assay was performed in solid medium plates according to the procedure described by Narváez-Barragán et al. (2020). The super optimal broth with 2% glycerol (SOBG) (Chen et al., 2016) contained per liter: 20 g tryptone, 5 g yeast extract, 10 mM NaCl, 2.5 mM KCl, 10 mM MgSO4, and 15 g agar supplemented with 2% glycerol. A single pure bacterial colony was inoculated at the center of solid SOBG plates using a toothpick. Plates were incubated for 3 days at 28°C. The width of the line was measured to calculate extracellular polysaccharide (EPS) production. Each treatment was carried out three times, and the assays were repeated three times.

#### Biofilm Formation and Quantification Assays

As described by Chen et al. (2016), the biofilm assay was performed in SOBG with minor modifications. An overnight bacterial culture was diluted at 1:100 with SOBG broth (SOBG medium without agar); 100 μl of the culture was dispensed into each well of 96-well microtiter plates and incubated at 28°C in an orbital incubator shaker (200 rpm) for 18 h. Then, bacterial cultures were removed and 125 μl of 0.1% crystal violet (w/v) were added. After 15 min of staining at room temperature, dye was washed three times with water. Stained wells were decolorized with 200 μl 95% ethanol after drying, and the attached bacterial cells were quantified. The concentration of crystal violet was determined by measuring the absorbance at 600 nm using a BioTek Epoch Microplate Spectrophotometer (Winooski, VT, United States).

#### Pathogenicity Assays

Pathogenicity assays were performed on taro corms and pineapple leaves to compare the novel strains with the type strain, NCPPB 2538 (=A5422; CFBP 2052). Taro corms and pineapple leaves were washed thoroughly under running water, surface sterilized with 0.6% sodium hypochlorite for 10 min, transferred and submerged in sterile water for 10 min, and dried inside the laminar flow hood. Taro corms and pineapple leaves were inoculated by making a small wound in the epidermis and placing 50 μl of a bacterial culture (OD_600_∼1.2) grown overnight in NBG (nutrient broth + 0.4 w/v% glucose) over the wound. As controls, taro corms and pineapple leaves were lightly wounded with a scalpel, and 50 μl NBG was placed over the wound. Corms and leaves were incubated for 48 h in a moist chamber at 25°C. Following incubation, taro tissues were weighed (g) without drying to determine the degree of tissue maceration. Each treatment was performed three times and each experiment was repeated three times.

### Data Analyses

All experiments were performed with three replicates. An analysis of variance was calculated using the IBM SPSS Statistics V25 (IBM Corp. Released 2017. Version 25.0. Armonk, NY, United States) one-way ANOVA (*p* < 0.05). Mean value differences were calculated by the Tukey’s test.

## Results

### Comparative Genomics and Phylogenomic Analyses Within the Genus *Dickey*a

Average nucleotide identity and *in silico* dDDH analyses showed that five *D. zeae* genomes were distinct from the other well-characterized species (Supplementary Figure 1). High genome dissimilarity was observed among the *D. zeae* strains with a dDDH value of 56–68.20%, which is lower than the recommended cut-off value for species (70%) (Wayne et al., 1987; Goris et al., 2007). Additionally, the ANI value within the *D. zeae* strains was 94.33–96.27%, which overlaps the threshold value for species (ANI 95–96%) (Goris et al., 2007; Richter and Rosselló-Móra, 2009; Kim et al., 2014; Chun et al., 2018; Wang et al., 2020). dDDH and ANI analyses indicated that the *D. zeae* strains shared the highest DNA homology with *D. chrysanthemi* Ech1591 (dDDH 30.40–30.9%; ANI 87.24–87.59%). ANI and dDDH values between *Dickeya* and *Pectobacterium* were 83.68–84.48% and 20.70–21.60%, respectively (Supplementary Figure 1). The dDDH phylogenetic tree was inferred with FastME 2.1.6.1 from the GBDP distances calculated from genome sequences (Meier-Kolthoff et al., 2013). Various strains of *D. zeae* were clustered, and *D. chrysanthemi* was the closely related species in a phylogenetic tree (Supplementary Figures 2A,B). Interestingly, while *P. atrosepticum* and *D. paradisiaca* were grouped according to ANI-NJ phylogenetic analyses, *P. atrosepticum* was an out-group strain in the dDDH phylogenetic analysis (Supplementary Figures 2A,B).

**FIGURE 2:**
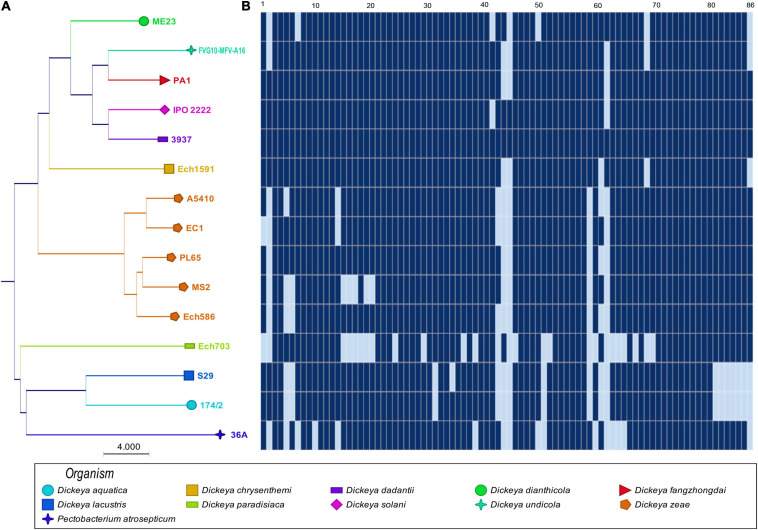
Sharing of plant cell wall-degrading enzymes (PCWDEs) in *Dickeya* species. The types of cell wall-degrading enzymes are indicated for each of the strains. **(A)** Concatenated Neighbor-Joining phylogenetic dendrogram based on 86 PCWDEs in *Dickeya* sp. The Neighbor-Joining method was applied to determine the distances, with each node being supported by a bootstrap of 1,000 replicates to assess reliability. **(B)** Color scales based on the presence and absence of 86 plant cell wall-degrading genes in the genomes. Dark blue (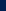) shows the presence of genes, and light blue (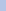) shows the absence of genes. In **(B)**, numbers 1–86 are described in Supplementary Table 1.

The phylogenetic analyses based on the concatenated sequences of 86 virulence-associated genes (Supplementary Table 1) were performed to evaluate the phylogenetic diversity within virulence-associated genes among the *D. zeae* strains and other members of *Dickeya*. The phylogenetic tree revealed two main separate clades: the first clade (Figure 2) grouped *D. zeae* strains with *D. dianthicola, D. undicola, D. fangzhongdai, D. solani, D. dadantii*, and *D. chrysanthemi*; the second clade consisted of *D. paradisiaca, D. lacustris, D. aquatica*, and *P. atrosepticum*. In the first clade, two major clusters were distinguished with one of these represented by *D. dianthicola, D. undicola, D. fangzhongdai, D. solani, D. dadantii, D. chrysanthemi*, *and D. zeae.* Five *D. zeae* strains are clustered on a separate branch. On the other hand, the second clade consisted of *D. paradisiaca, D. lacustris, D. aquatica*, and *P. atrosepticum.*

### The BLAST Matrix Analysis in the Genus *Dickeya*

**FIGURE 3:**
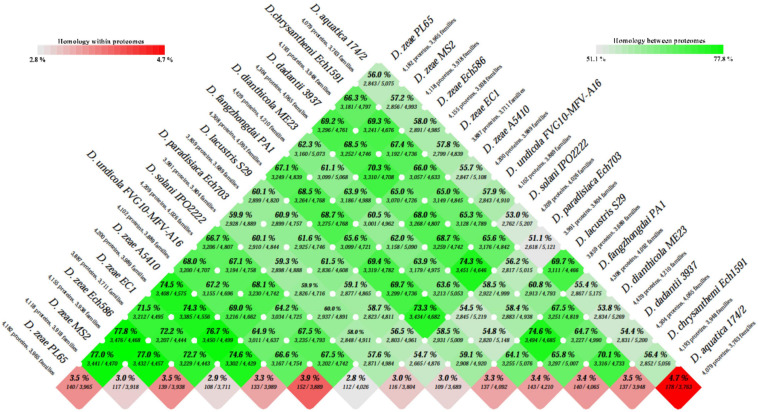
Basic Local Alignment Search Tool (BLAST) matrix between and within the total proteomes of *Dickeya* genus. A pairwise protein comparison was performed using BLAST. All protein-coding sequences were compared with each other across the genomes. The BLAST hit was considered as significant when 50% of the alignment showed identical matches and if the coverage of an alignment was at least 50%. The color scale intensity from dark green to light green highlights a decrease in the degree of homology between the proteomes, whereas the color scale from dark red to light red shows decreasing homologous hits within the proteome itself.

The BLAST matrix heatmap was generated to determine the similarity in each of the conserved protein families present within the 14 *Dickeya* species. A pairwise comparison of total protein-coding genes among the 14 *Dickeya* genomes ranges from 51.1 to 77.8% of the shared proteins, with the lowest value representing the pairwise identity between *D. aquatica* and *D. paradisiaca* and the highest between PL65 and Ech586 *D. zeae* genomes (Figure 3). The number of proteins and protein families used to compare all proteomes was the lowest for the genome EC1 of *D. zeae* with 3,887 proteins and 3,711 families (Figure 3). The proteome comparison displayed that the average protein family similarity among *D. zeae* genomes ranges from 71.5 to 77.8%. In comparison, the intra-proteome homology among the protein families within each genome is less than 3.5% (Figure 3). These results indicated that *D. zeae* is genetically distinct from other species. The blast matrix results are concordant with ANI and dDDH analyses. The BLAST matrix results also demonstrated that *D. zeae* proteomes are diverse, with an average of 74% sequence identity, one exception was the sequence identity of 77.8% between Ech586 and PL65 genomes; this could be due to the isolation of these strains from the hosts of the Araceae family (*C. esculenta*-PL65 and *Philodendron* Schott-Ech586). Overall, these results suggest that there was a significant diversity among the strains of *D. zeae*.

### Genomic Evolution of *Dickeya* Species Based on the Analysis of Codon and Amino Acid Usage

A single amino acid can be generated by more than one codon, termed as synonymous codons (Uddin, 2017). The codon usage shows a variation among genomes of various species. Hence, the codon usage pattern establishes a unique characteristic of each species (Uddin, 2017). Hence, we analyzed and contrasted a bias in the third codon position, and amino acid frequencies for the 14 *Dickeya* species. The corresponding analysis consisted of quantifying the fraction of each codon and amino acid count of the total number of codons and amino acids. The percentage of codon and amino acid profiles among the species was calculated and visualized in heatmaps (Supplementary Figure 3). The codon usage heatmap displayed an intense yellow color for the usage of GC-rich codons like GCG, CGC, CTG, CAG, CGG, CCG, GGC, and GCC heatmap (Supplementary Figure 3A). The amino acid usage heatmap revealed that amino acids like alanine (A), arginine I, leucine (L), and serine (S) (indicated as pink color scales) were used in a higher frequency in the *Dickeya* species (Supplementary Figure 3B). The intensity of color gradually changed from pink to blue when the amino acid frequency is increased. The clades were distinct in generated phylogenetic patterns. Strains of *D. zeae*, *D. undicola, D. aquatica*, and *D. lacustris* were grouped in the same clade while the remaining species formed distinct lineages. Interestingly, *D. chrysanthemi* was phylogenetically distinct from all strains of *D. zeae* but the genomes of *D. paradisiaca* and *D. chrysanthemi were* grouped. Furthermore, in contrast to previous phylogenetic analyses, the amino acid and codon dendrograms displayed different relationships within the *Dickeya* species.

### The Pan and Core Genome Analysis in the Genus *Dickeya*

**FIGURE 4:**
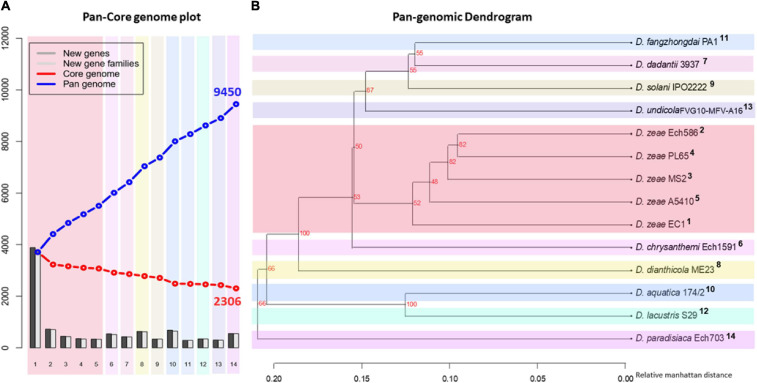
Pan- and core-genome analyses among the *Dickeya* species. Pan- and core-genome plot: **(A)** The pan and core genome analyses were developed by employing BLAST with a cutoff of 50% identity and 50% coverage of the longest gene. **(B)** Pan-genome tree: the respective dendrogram illustrates the grouping of species based upon the shared gene families (core genome) defined in the pan- and core-genome analysis. Organisms are marked 1–14; 1, PL65; 2, A5410; 3, MS2; 4, Ech586; 5, EC1 (*D. zeae*); 6, *D. undicola* FVG10-MFV-A16; 7, *D. aquatica* 174/2; 8, *D. lacustris* S29; 9, *D. solani* IPO 2222; 10, *D. dadantii* 3937; 11, *D. dianthicola* ME23; 12, *D. fangzhongdai* PA1; 13, *D. paradisiaca* Ech703; and 14, *D. chrysanthemi* Ech1591.

To complement our previous analysis and discover similarities in general genomic content, a pan-core genome analysis was carried out using the 14 *Dickeya* genomes. A pan-core genome plot with the corresponding calculated output is presented in Figure 4A. A final core genome of 2,306 gene families and a pan-genome of 9,450 gene families were obtained among 14 *Dickeya* genomes. The addition of genomes in the analysis caused a decrease in the core genome size, indicating genome heterogeneity among the 14 *Dickeya* species. Considering the average gene number of ∼4,720 for the *Dickeya* genomes, 2,306 core genes represented approximately 50% of the total genome. Approximately half of the genomic constituents were conserved or orthologous across the 14 genomes analyzed in this study. The high pan-genome size, which is more than three times the core size, seems to suggest a significant genetic variation among the *Dickeya* species. The *D. zeae* strains showed a similar pattern of core- and pan-genome size variation in Figure 4A, indicating the heterogeneity within this complex group. To understand the genome-based relationships across the 14 *Dickeya* species, a phylogenetic tree was constructed using 2,306 conserved genes. The generated dendrogram formed a clear clade for the *D. zeae* strains (Figure 4B). *Dickeya chrysanthemi* was close to the group of *D. zeae* strains. Meanwhile, *D. aquatica* and *D. lacustris* were grouped.

### Comparison of Main DNA Features Among the *D. zeae* Complex Strains Isolated From Distinct Hosts

**FIGURE 5:**
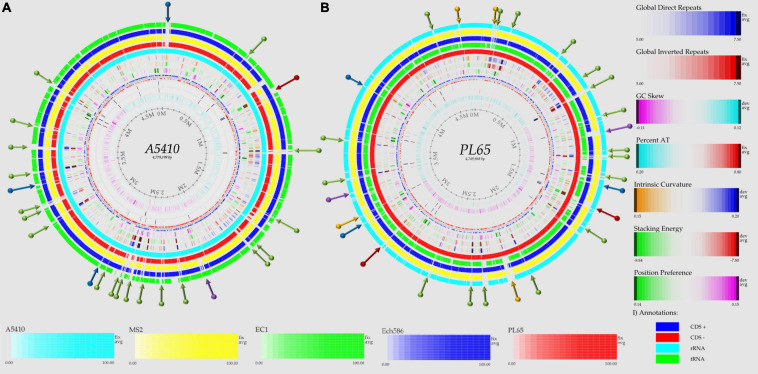
Genome BLAST atlases within the *D. zeae* complex species. The genomes of novel strains **(A)** A5410 and **(B)** PL65 were used as references to generate the circular graphics. Data of DNA, RNA, and gene features of both the references were obtained after annotating the genomes using the NCBI prokaryotic genome annotation pipeline (PGAP). From the most inward lane, the figures display the size of the genome (axis), percent AT (red = high AT), GC skew (blue = most Gs), inverted and direct repeats (DRs; color = repeat), a position preference, stacking energy, and an intrinsic curvature. Following these layers, the external solid rings (indicated with a unique color) represent the genomes of other *D. zeae* strains mapped against the references. Olive arrows highlight those unique DNA regions associated with a high intrinsic curvature, stacking energy and a position reference found solely in the novel strains A5410 and PL65. Dark-red arrows pinpoint the areas of the genome with a low intrinsic curvature, stacking energy, and a position reference. Dark-blue arrows indicate a low intrinsic curvature and low stacking energy but a high position reference. Orange arrows show areas of the genome displaying a high intrinsic curvature and high stacking energy but a low position reference, whereas purple arrows represent those genetic zones absent in some *D. zeae* isolates. BLAST genome atlases were created using the CMG-biotools pipeline.

To evaluate and compare the genomic properties of strains A5410 and PL65 sequenced in this study, BLAST atlases were created using these two genomes as references and compared with the other *D. zeae* complex strains EC1, Ech586, and MS2. The main DNA features, namely genome size, percent AT (red indicates high AT), GC skew (blue indicates most G’s prevalence), direct and inverted repeats (blue and red, respectively), position reference, stacking energy, and intrinsic curvature, were drawn in the atlas for each reference. Following these layers, all genome queries were displayed in the atlases as specific-colored lines, where only the gene regions that matched with the reference organism were drawn. Several notorious divergences in the intrinsic genomic features of these two new strains were observed with respect to the other *D. zeae* strains. Strain A5410, for instance (Figure 5A), harbored 11 inverted and 20 direct repeats (DRs), and among them, 10 DRs were exclusive to this strain. Regarding the DNA properties, the strains A5410 (pineapple host) and PL65 (taro host) and Ech586 contained a genomic region that displayed a low intrinsic curvature, low stacking energy, and a low position reference (pinpointed in a dark red arrow). Moreover, the three regions of a low intrinsic curvature, low stacking energy but a high position reference (pinpointed with a dark blue arrow) were only found in the pineapple strain A5410. Another gene zone with the same DNA features was absent in strains MS2 and Ech586 (pinpointed with a purple arrow).

On the other hand, the taro strain PL65 exhibited 12 and 22 inverted and direct global repeats, respectively (Figure 5B), of which 6 DRs were not found in the other isolates. This strain was the only one that presented three DNA regions with a low intrinsic curvature, low stacking energy but high position reference features (pinpointed with a blue arrow). The other two regions with similar features (indicated by a purple arrow) were also observed; one of these regions was not observed in the genomes of strains A5410 and EC1 while the other was absent in MS2 and Ech586. Additionally, the strain PL65 exhibited four characteristic regions that displayed a high intrinsic curvature and high stacking energy but low position reference values (orange arrows). These regions were not visualized in the other strains. Lastly, a total of 17 gene regions with the same features mentioned previously but with a high position reference were shown by the genome of PL65 but not in the other organisms. Altogether, the DNA properties analyzed in these two new strains clearly demonstrated a high divergence in the genome properties among the *D. zeae* strains.

### General Genomic Features of Two Novel Strains (PL65 and A5410)

The depth (X) of the assemblies was 558 and 701 for A5410 and PL65 strains, respectively. The complete genomes of novel strains, A5410 and PL65, consisted of a single circular chromosome of 4,779,199 and 4,749,968 base pairs, with a GC content of 53.5% and 53.6%, respectively. The genome PL65 contains 4,182 protein-coding DNA sequences (CDS), 75 transfer RNA- (tRNA-) coding genes, 22 ribosomal RNA-coding (5S-16S-23S) genes, nine non-coding RNA genes, and 87 pseudogenes. The genome A5410 contains 4,305 protein-coding DNA sequences (CDS), 75 tRNA-coding genes, 22 ribosomal RNA-coding (5S-16S-23S) genes, eight non-coding RNA genes, and 90 pseudogenes. Detailed information of five genomes is provided in Supplementary Table 2. The length of *D. zeae* genomes were in a range between 4.2 and 4.3 Mb. The GC content percent was almost similar (∼53%) for all five genomes. Regarding total conserved protein-coding sequence genes (CDS), the A5410 *D. zeae* genome displayed the highest CDS with 4,305 genes and the highest pseudogenes with 90 genes (Supplementary Table 2).

**FIGURE 6:**
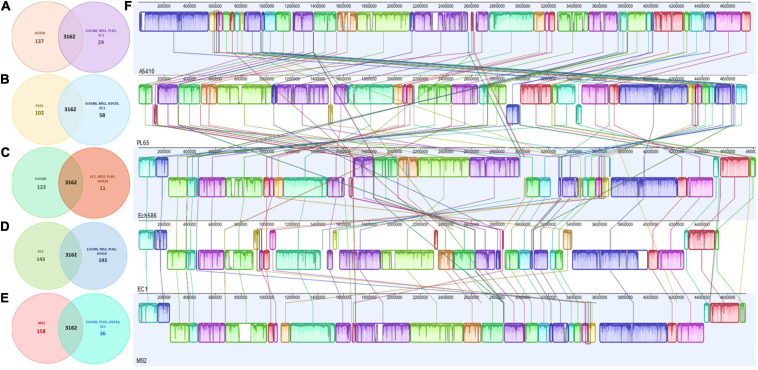
Comparison of *D. zeae* genome sequences against each other. Venn diagram showing the number of clusters of orthologous genes, which are shared and unique at the strain level. Venn diagrams **(A–E)** are shown for the deduced proteins of A5410, PL65, Ech586, EC1, and MS2, respectively. Values were calculated by OrthoMCL clustering analyses using the following parameters: *p*-value cut-off = 1 × 10^–5^; identity cut-off = 90%; percent match cut-off = 80, deduced proteins of A5410, PL65, Ech586, EC1, and MS2, respectively. **(F)** The complete genome alignment of five linearized *D. zeae* genomes was performed using progressiveMAUVE. The scale represents the coordinates of each genome. Different color blocks represent local collinear blocks (LCBs), which are conserved segments in five genomes. Within LCBs, the white area represents low similarity regions or regions unique to one genome but absent in another. LCBs above the black horizontal central line are in forwarding orientation and below this are in reverse orientation. Colored lines show the rearrangement of LCBs among the genomes.

OrthoMCL pipeline was used to develop a robust comparative genomics analysis. The results showed that the number of core genes among the five genomes were 3,162 genes. The number of specific genes in A5410, PL65, Ech586, EC1, and MS2 were 137, 102, 123, 143, and 158, respectively (Figures 6A–E). In Figure 6F, the color and orientation of local collinear blocks (LCBs) indicated a high diversity among the *D. zeae* genomes.

**FIGURE 7:**
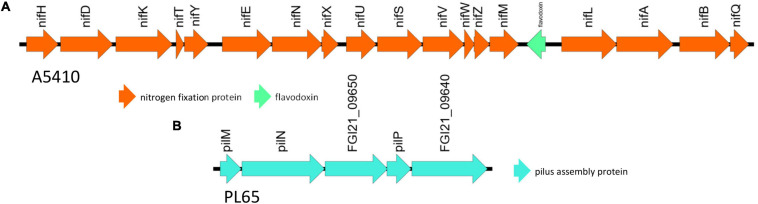
The unique gene clusters present in *D. zeae* strains. **(A)** The nitrogen fixation cluster from genome A5410. **(B)** The pilus assembly protein cluster from PL65.

**FIGURE 8:**
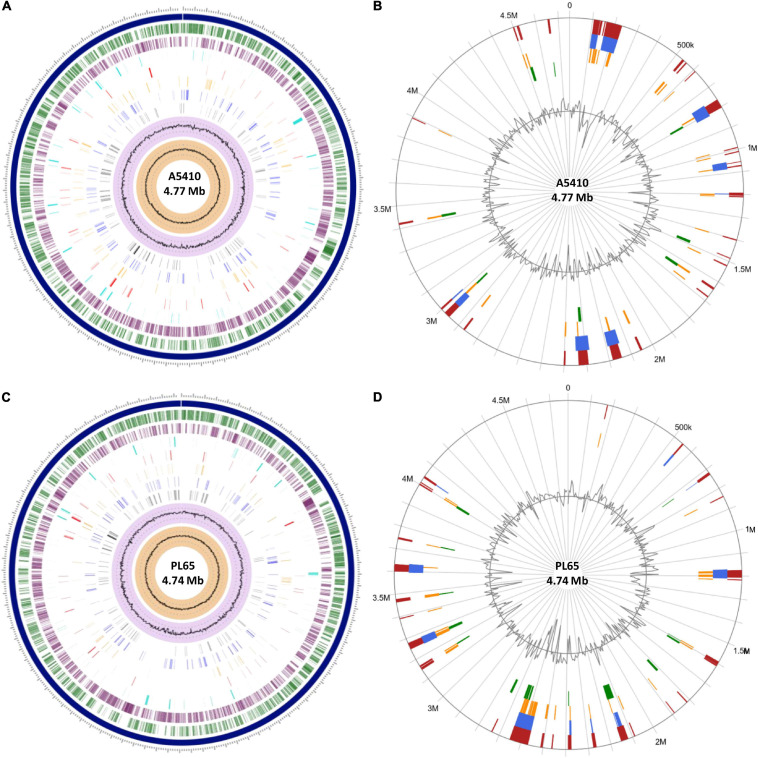
The circular view of the genomes of **(A)** A5410 and **(C)** PL65 strains generated using Proteome Comparison tool of Pathosystems Resource Integration Center (PATRIC) showing the physical map of significant features. From outside in: position label Mb (shown in 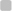); the order of contigs (shown in 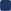); distribution of coding sequences in forward strands (shown in 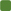); distribution of coding sequences in reverse strands (shown in 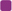); distribution of non-coding elements along the chromosome (shown in 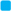); distribution of genes involved in antibiotic resistance (shown in 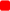); distribution of other virulence factors (shown in 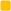); distribution of genes encoding transporter proteins (shown in 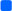); distribution of genes encoding drug targets (shown in 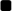); distribution of GC content (shown in 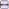); and distribution of GC skew (shown in 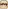). Circular visualization of the predicted Genomic Islands (GIs) on A5410 **(B)** and PL65 **(D)** strains. The analysis was conducted in IslandViewer 4. The interactive visualization of the distinct islands across the genomes is shown with blocks colored according to the predictor tool as described: IslandPick (shown in 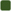) based on genome comparison, IslandPath-DIMOB (shown in 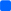) based on associated GIs features such as transfer RNAs (tRNAs), transposon elements, integrases, and sequence bias, SIGI-HMM (shown in 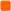), based on the codon usage bias with a Hidden Markov model criterion and the integrated results of the four tools (shown in 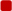).

The genomes of two novel strains were analyzed extensively. The genome A5410 harbored some important unique genes, such as transporter proteins, endonuclease protein, phage tail protein, aspartate/glutamate racemase family protein *arsD* gene involved in arsenic resistance (Firrincieli et al., 2019), and the carboxymuconolactone decarboxylase protein involved in protocatechuate catabolism (Cheng et al., 2017). The nitrogen fixation gene cluster was only present in the A5410 strain isolated from pineapple (Figure 7A). The genome PL65 harbored some important unique genes, such as glycosyltransferase protein, transporter proteins, endonuclease protein, and phage tail protein. The pilus assembly protein cluster was only present in the PL65 genome isolated from taro (Figure 7B). Significantly, the pilus cluster was predicted within a GI (Figure 8). GIs or horizontal acquired islands (HAIs) are incorporated into the bacterial genome during the conjugation process, and besides, harbor genes are required for integration and excision into the chromosome (Johnson and Grossman, 2015; Zakharova and Viktorov, 2015). The genomes of PL65 and A5410 were screened for horizontally acquired DNA using IslandPath-DIMOB, SIGI-HMM, IslandPick, and Islander methods integrated with the IslandViewer server (Bertelli et al., 2017; Figure 8). In the genome of A5410, 56 presumed genome islands (GIs) ranging from 2.6 to 83.1 kb were detected. The largest GIs consisted of 83,100 bp with 92 predicted gene coding regions, whereas the shortest GIs consisted of only 5 predicted gene coding regions. A total of 707 genes were predicted into the GIs. Only 68 open reading frames (ORFs) were unique genes for A5410 among the analyzed genomes. For strain PL65, 47 presumed GIs ranging from 3.1 to 70.3 kb were detected, of which the largest consisted of 70,338 bp and predicted to encode 69 genes. A total of 675 genes were predicted into the GIs. Only 55 ORFs were unique genes for PL65 among the analyzed genomes. Genes encoding tRNAs, transposases and integrases, Pel, endoglucanase, and phage tail protein and genes related to the citrate synthase system, the T6SS (*vgrG*), the T4SS (Rhs), toxin–antitoxin (TA) systems, and antibiotic biosynthesis were identified in the GIs of both strains. Furthermore, genes related to flagella and chemotaxis, type IV pilus biogenesis system, CRISPRcas1, CRISPRcas2, and type III CRISPRCsm1-6 were identified in the Gis of the strain PL65.

### Genome Comparison of Gene Clusters Associated With Pathogenesis Among the *D. zeae* Complex

The soft rot bacteria within the genus *Dickeya* macerate the plant tissue and acquire nutrients from the dead cells (Davidsson et al., 2013). Several pathogenicity determinant genes are involved in this process. Most pathogenicity determinants genes, including the PCWDE, type secretion systems (types I–VI), the synthesis of polysaccharides (ECA, CPS, LPSs, exopolysaccharides, and O-antigen), bacterial attachment operons (type IV pili), flagella and chemotaxis, quorum-sensing systems and zeamine synthesis, have been described for EC1 strain (Zhou et al., 2015). We analyzed and compared the similarities, differences, or absence of virulent determinant genes among the *D. zeae* complexes. Studies regarding the genome of EC1 strain were carried out.

#### Plant Cell Wall-Degrading Extracellular Enzymes and Proteases

The PCWDE, including pectinases, Peh, cellulases, and Prts, are essential virulence determinants, which degrade the structural components of the plant cell wall, playing a significant role during bacterial pathogenesis and disease development (Toth et al., 2006; Nykyri et al., 2012; Davidsson et al., 2013; Charkowski et al., 2014). The PCWDE are the main responsible factors in creating soft rot symptoms.

Most of these pectinases—Pel, pectin lyase (Pnl), pectin methyl esterase (Pme), and Peh—are scattered throughout the genomes rather than arranged in clusters (Glasner et al., 2011; Nykyri et al., 2012). The pectinases encoded by independent genes seem to be derived from the successive rounds of gene duplication (Barras et al., 1987; McMillan et al., 1994). The *pelABCDEILNZ* genes encoding endo-Pels and *pelW* and *pelX* genes encoding pectate disaccharide-lyases were highly conserved in *D. zeae* genomes. Similarly, *pemAB* genes encoding Pem were conserved in *Dickeya* species.

*pehKNVWX* genes encoding Peh were also conserved in *Dickeya* species. Moreover, the *D. zeae* Ech586, A5410, and PL65 indicated the loss of *pnl*GH genes encoding Pnl (Supplementary Table 1 and Supplementary Figure 2). All species harbored the Paes (*paeX* and *paeY*). The *Ogl* was present in all analyzed *Dickeya* species. The *ganABCEFGKLR* gene cluster responsible for removing galactan chains in pectin-ramified regions were present in all *Dickeya* species (Hugouvieux-Cotte-Pattat et al., 2014). However, the MS2 strain of *D. zeae* lost the *ganABCEFG* genes (Supplementary Table 1 and Supplementary Figure 2). The rhamnogalacturonate lyase *rhi*E gene was present in all *D. zeae* strains. The ferulate esterase *faeD* and *faeT* genes were present in all *Dickeya* species except *D. zeae* EC1 and A5410 in which the *faeT* gene was lost. The regulator *Kdg* was present among the *Dickeya* species. Genes *exuRT, kduDI, uxaABC*, and the transporter *togABMNT* were highly conserved and present in all tested genomes (Supplementary Table 1 and Supplementary Figure 2).

#### Cellulases and Xylanases Enzymes

The endoglucanase genes (*celY* and *cel*Z), beta-glucosidase encoding genes (*bglA*, *bgxA*, *bglB*, *nagZ*, *bglC*, *bglD*, and *celH*), and an alpha-glucosidase encoding gene (*lfaA*) are involved in the degradation of Cel to glucose (Zhou et al., 2015). Although these genes were conserved in the genus *Dickeya*, the *bglC* gene was absent in some *D. zeae* genomes (MS2, Ech586, and A5410), *D. lacustris* S29, and *D. aquatica* 174/2 (Supplementary Table 1 and Supplementary Figure 2). The xylanases (*xynA*) gene is involved in the degradation of xylan and xyloglucan, mainly present in plant cell walls (Pena et al., 2016). *D. zeae* genomes (A5410, PL65, EC1, Ech586, and MS2) contained *xynA* (Supplementary Table 1 and Supplementary Figure 2).

#### Protein Secretion Systems

The T1SS constitutes *prtD, E*, and *F*, and is responsible for secreting the Prts. The *prt* cluster encodes four metalloproteases (PrtABCG) and three Prt secretion-associated proteins (PrtDEF) (Zhou et al., 2015). The metalloprotease PrtW plays an important role in degrading the plant cell wall proteins (Charkowski et al., 2012; Zhou et al., 2015). On the other hand, the T2SS is responsible for translocating extracellular proteins across the outer membrane (Jha et al., 2005; Zhou et al., 2015). The *out* cluster encodes two outer membrane proteins (OutSD), five inner membrane proteins (OutBEFLM), one trans periplasmic protein (OutC), and prepilin peptidases (OutGHIJLK) (Zhou et al., 2015). T1SS (within the range of 66–100% nucleotide identity) and T2SS (within the range of 82–100% nucleotide identity) were present and highly conserved in the *D. zeae* genomes (Locus tag of T2SS; EC1: W909_RS13390-RS13455, Ech586: DD586_RS13945-RS14010, MS: C1030_RS14365-RS14430, A5410: FGI04_04420-04355, and PL65: FGI21_21175-21110. Locus tag of T1SS; EC1: W909_09760-09795, Ech586: DD586_2059-2052, MS: C1030_10785-10820, A5410: FGI04_08210-08175, and PL65: FGI21_03340-03305) (Supplementary Figure 4).

**FIGURE 9:**
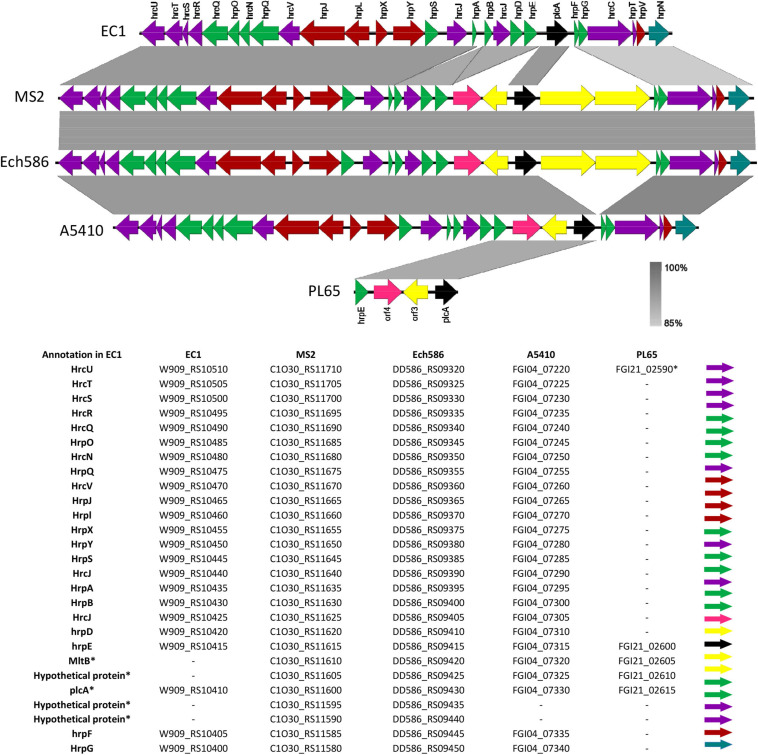
Comparison of the genetic organization of type III secretion system (T3SS) among five *D. zeae* strains. The arrow position represented a forward/reverse gene orientation. The arrow color signified a specific gene composition within the T3SS. A pairwise alignment between the linear sequences was rendered based upon the BLAST algorithm with cut-off values from 85 to 100%. Regions with a higher nucleotide identity were displayed with a shaded gray.

The T3SS is integrated by the hypersensitive response and pathogenicity (*hrp*) and hypersensitive response conserved (*hrc*) gene clusters (Toth et al., 2006; Zhou et al., 2015). According to our analyses, T3SS was present and highly conserved within a range of 85–100% nucleotide identity in the genomes of *D. zeae* complex (EC1, MS2, Ech586, and A5410), except PL65 (Locus tag of T3SS; EC1: W909_RS10510-RS10380, Ech586: DD586_RS09320-RS09470, MS: C1030_RS11710-RS11560, A5410: FGI04_007220-07360, and PL65: FGI21_02600-02615) (Figure 9). Most genes of T3SS were absent in PL65 except for the *hrpE* (FGI21_02600) and *hrpU* (FGI21_02590) genes. While the size of *hrpU* was 1,080 bp, the *hrpU* gene of PL65 is only 158 bp, with an average 94% identity. In the phytopathogen *Xanthomonas citri* subsp. *citri*, the HrpE protein, encoded by *hrpE*, was discovered as an elicitor of plant defense responses and described as a primary structural component of T3SS (Gottig et al., 2018). The *D. zeae* (EC1, MS2, Ech586, and A5410) genomes harbored a large gene cluster with three transcriptional units identified, spanning a genomic region of ∼25 kb. The genetic region of T3SS was between the *hrpN* and *hrcU* genes within the genomes of *D. zeae* complex. The *plcA* gene, which encodes an extracellular phospholipase, was present in all *D. zeae* genomes. The *hrp* and *hrc* gene clusters and the near upstream region of the *plcA* gene were highly conserved. While there were no additional genes in the EC1 genome, MS2, and Ech586 genomes harbored four extra ORFs encoding three hypothetical proteins and a membrane-bound lytic murein transglycosylase *MltB*. Additionally, A5410 and PL65 genomes harbored two ORFs encoding one hypothetical protein and *mlt*B gene (Figure 9).

Type IV secretion system constitutes *virB1-11* genes and functions in conjugation, pathogenicity, and DNA release/uptake (Bhatty et al., 2013; Chandran Darbari and Waksman, 2015; Trokter and Waksman, 2018). Some *D. zeae* strains (EC1, Ech586, and A5410) harbor virB-T4SS cluster homologs to *Agrobacterium tumefaciens;* similarly, some *Pectobacterium* species also contain virB-T4SS cluster (Arizala and Arif, 2019). The T4SS gene cluster is highly conserved within the range of 91–100% nucleotide identity among EC1, Ech586, and A5410, but not found in PL65 and MS2; both strains lacked most of the T4SS genes and only had *virB1* and *virB2* (Supplementary Figure 5).

The type V secretion system (T5SS) encompasses either one or two proteins, the latter constituting two-partner secretion systems called *Hec*/*Tps*/*Cdi* (contact-dependent inhibition) (Charkowski et al., 2012; Pédron et al., 2014). In the *D. zeae* genomes, just one *Hec*/*Tps*/*Cdi* system was present and harbor one *hecB* and one *hecA* genes that have been shown to act in contact-dependent growth inhibition (CDI) by delivering the C-terminal toxin domain of *HecA* (*TspA*/*CdiA*) to target cells (Pédron et al., 2014). The genes *hecA*2 and *hecB* (T5SS) were located near the T3SS in all strains of the *D. zeae* complex. The hemagglutinin-coding loci *hecB* and *hecA* were present within the range of 68–100% nucleotide identity among the *D. zeae* complex strains (Locus tag of T5SS; EC1: W909_RS10375, W909_RS19830, Ech586: DD586_RS09475-RS22000, MS: C1O30_RS11550-RS11555, A5410: FGI04_07365-07405, and PL65: FGI21_02620-02625) (Supplementary Figure 6A). All *D. zeae* genomes harbored a *hecB* homolog. The *hec* system was universal among these *D. zeae* genomes. However, we found that the gene sequences encoding *hec*A2 in some *D. zeae* genomes were truncated. For instance, the gene sequences encoding *hec*A2 in Ech586 and A5410 were incomplete, and the entire system was annotated as pseudogenes. This finding indicated that this cluster might not be functional in these genomes. Besides, we observed the inserted genes in A5410 and Ech586 genomes. One hypothetical protein and one type I TA system protein (SymE) were located between the first and second ORF for HecA protein in the Ech586 genome. Additionally, A5410 harbored six extra genes: three hypothetical genes, two SymE, and one membrane channel gene—between incomplete *hecA* genes. HecA protein has two ORFs, and between those ORFs, three hypothetical proteins were located, two type I TA system protein (SymE) and one IS3 (insertion sequences) transposase. The genome of EC1 harbored *hecA* gene; however, *hecA* exhibited a 50% query cover and 94% identity with PL65 and MS2 genomes.

The T6SS targets other bacteria and thus plays an essential role as a polymicrobial injectosome that resembles a bacteriophage tail (Bernard et al., 2011). The T6SS assists in multiple biological processes, for instance, an interaction with host eukaryotic cells, pathogenicity, antibacterial activity, symbiosis, metal ion acquisition, and biofilm formation (Bernard et al., 2011; Cianfanelli et al., 2016; Gallique et al., 2017). Regarding the genomes of *D. zeae* complex, the T6SS cluster was confirmed by 17 genes. The T6SS consists of the *hcp*, *vgrG* (virulence-associated protein G), *impBCF*, and *vasABCDEFGHIJKL* genes. The T6SS genes were highly conserved within the range of 68–100% in EC1, MS2, Ech586, A5410, and PL65 (Locus tag of T5SS; EC1: W909_RS06255-RS06425, Ech586: DD586_RS06380-RS06525, MS: C1O30_RS06775-RS06960, A5410: FGI04_12385-12215, and PL65: FGI21_07465-07265). Additionally, 20 genes on an average were inserted into the T6SS cluster in all five genomes (Supplementary Figure 6B). We found substantial variations in an extra set of genes inserted between *vgrG* and *imp*B (Supplementary Figure 6B). The inserted cluster was annotated as ankyrin genes, hypothetical proteins genes, the repeat-containing protein rearrangement hot spot (*rhsA*s), amidohydrolase gene, *symE* genes, and *parDE* genes.

#### Flagellar and Chemotaxis Genes

The flagellar biosynthesis and chemotaxis clusters constitute 20 *fli* genes (*Fli*Z*ACD,T*), 14 *flg* genes (*flgA,N*), and 5 *flh* genes (*FlhA,E*), involved in the flagellar synthesis, four flagellar rotation genes (*motAB*, MCP1, and MCP3) and six chemotaxis-associated genes (*cheABZYRW*) (Zhou et al., 2015). The flagellar biosynthesis and chemotaxis genes were present and highly conserved in *D. zeae* genomes (Supplementary Table 1). Interestingly, the EC1 genome harbored two sets of *fli*C genes. There are 12 additional genes inserted between *fliA* and *fliC* in EC1, Ech586, and MS2 genomes. These inserted genes consisted of two methyltransferases (*rfbC* and *fkbM*), an aminotransferase (*spsC*), and nine fatty acid biosynthesis genes (*aldH*, *luxE*, *fadD*, *tktAB* two sets of *fabG*, *acpP*, and *maa*). Moreover, A5410 and PL65 also contained six inserted genes (*rfbC*, *mocA*, two sets of *fkbM* and *carA*), which were highly conserved for A5410 and PL65 genomes (Supplementary Table 1).

#### Twitching Motility Genes

The type IV pilus biogenesis encoding system consists of *pilF* (type IV pilus biogenesis and stability protein), *pilT* (twitching motility protein) and *pilABC*, and *pilM-Q*genes (Maier and Wong, 2015; Duprey et al., 2019). The *pilF* and *pilT* genes were located distant from the type IV pilus biogenesis cluster. The type IV pilus biogenesis system is present and highly conserved in the *D. zeae* genomes (Supplementary Table 1).

#### Polysaccharide Genes

The ability to biosynthesize polysaccharides, which can be secreted as EPS or remain attached to the bacterial cell surface (LPS and CPSs), is another important factor in infection (Whitfield, 2006). Bacteria display different types of polysaccharides, namely LPSs, which attach to the cell membrane, CPSs that bind covalently to the cell surface, lipo-oligosaccharides (LOS) that lack the O-antigen, and the EPS, which are secreted into the surrounding environment (Reeves et al., 1996). In our analysis, we observed that the CPS cluster was composed of 12 genes (*cpsABC*−*wcaB*), which were highly conserved in Ech586, MS2, PL65, and A5410. However, the entire cluster was absent in EC1 (Supplementary Table 1). The ECA is composed of 10 genes (*rffM*-*wecA*) that were present in all genomes (Supplementary Table 1). Interestingly, the genome MS2 harbors one inserted gene (hypothetical protein) between *wzzE* and *wecA* genes. The EPS cluster was composed of 22 genes (*gnd-wza*). All *D. zeae* genomes harbored the EPS cluster. However, EC1 and Ech586 had one extra glycosyltransferase protein located between *gnd* and *galF* genes. The genome Ech586, A5410, and PL65 harbored a highly conserved LPS cluster, which was encoded by 11 genes (*coaD*-*rfaD*) (Supplementary Table 1). In the case of the genome EC1, the LPS cluster showed a high number of rearrangements (Supplementary Table 1). The five genes of glycosyltransferase protein, including *rfaG* and *rfaQ*, were absent in EC1. However, the EC1 genome harbored additional six glycosyltransferase genes and three hypothetical genes. Unlike the others, the LPS cluster of EC1 presented 14 genes (Supplementary Table 1).

#### *D. zeae* Strains Contained Different CRISPR-Cas Systems

Most bacteria harbor the CRISPR-Cas immunity systems to protect themselves from foreign genetic elements (Makarova et al., 2011). CRISPR-Cas systems contain two groups, class 1 (types I, III, and IV), which includes an interaction with multi-Cas protein complexes, and class 2 (types II, V, and VI), which uses a single interaction effector protein (Hille et al., 2018). The CRISPR-Cas systems were identified in five *D. zeae* genomes using the CRISPRfinder online server (Grissa et al., 2007). *D. zeae* strains contained three types of CRISPR-Cas systems (subtype I-F, subtype I-E, and type III-A) (Supplementary Table 3 and Supplementary Figure 7). The genome of EC1, Ech586, MS2, and PL65 harbored the highly conserved subtype I-E CRISPR-Cas system composed of *cas3*, *casA*, *casB*, *cas7e*, *cas5e*, *cas6e*, c*as1e*, and *cas2* (Supplementary Table 3). The Ech586, MS2, A5410, and PL65 genomes presented the subtype I-F CRISPR-Cas system. The regular subtype I-F CRISPR-Cas system contains *cas1f*, *cas 3f*, *csy1*, *csy2*, *csy3*, and *cas6f* as found in MS2. However, Ech586, A5410, and PL65 confined a set of sequences downstream of the c*as*6f operon. These genes were identified as the coding sequences of the AsnC transcriptional regulator protein, YitT integral membrane protein, aspartate/tyrosine/aromatic aminotransferase protein, amino acid permease, and other hypothetical proteins. Interestingly, the A5410 genome harbored only the subtype I-F CRISPR-Cas. The type III-A CRISPR-Cas system was only found in PL65 and Ech586 genomes (Supplementary Figure 7). Meanwhile, the genome of PL65 harbored two hypothetical proteins between the genes *cms*3 and *cms*5 and the other between *cas*6 and CRISPR region. PL65 and Ech586 genomes harbored the type III-A CRISPR-Cas system. Finally, we observed the presence of orphan CRISPRs that were distant from the *cas* operon regions—EC1 contained two orphan CRISPRs loci, whereas Ech586, MS2, and PL65 possessed one orphan CRISPR. The orphan CRISPRs seems to operate far away from the *cas* locus, but they can be non-functional (Zhang and Ye, 2017).

The main characteristics of CRISPRs, such as position, length of DRs, number of spacers, and orientation, are provided in Supplementary Table 3. Generally, the length of DRs within all CRISPRs were 28–29 bp, except for the DRs (37 bp) of CRISPR located within the type III-A CRISPR-Cas system of PL65. We observed the shortest CRISPRs with a size of 302 bp—predicted for CRISPR5 in Ech586. The largest CRISPRs with a size of 3,022 bp—predicted for CRISPR2 in EC1. The highest number of 49 spacers were detected in the orphan CRISPR of EC1.

#### Secondary Metabolites Within the *D. zeae* Complex

The *D. zeae* complex revealed several genes involved in the synthesis of secondary metabolites. We used antiSMASH 4.0 server (Blin et al., 2017) to screen antimicrobials components. Four secondary metabolite biogenesis genes were predicted within the *D. zeae* complex genomes and are summarized in Supplementary Table 4. The five genomes harbor three well-known secondary metabolite biogenesis clusters (ind-vfm-expI, chrysobactin, and achromobactin), produced by *Dickeya*. The *ind-vfm-expI* genes are responsible for the synthesis of the indigoidine molecule and the quorum-sensing mechanism (Charkowski et al., 2012; Nasser et al., 2013). The chrysobactin and achromobactin genes are involved in the biosynthesis of siderophores (Reverchon and Nasser, 2013). Five strains also possess the gene cluster involved in the biosynthesis of cyanobactin-related molecules, which confers cytotoxicity. Further, seven clusters detected by AntiSMASH were found only in EC1, A5410, PL65, and Ech586; the bacteriocin synthesis cluster is present in all strains, except EC1. Beta-lactone-containing Prt inhibitor gene was predicted in all strains, except EC1. The arylpolyene biosynthesis cluster was identified in A5410, PL65, and Ech586. The bicornutin A1/A2 biosynthetic gene cluster (W909_RS19810, RS06850-RS06795), oocydin A biosynthetic gene cluster (W909_RS17185-17265), and a zeamine biosynthetic cluster (W909_RS19800, RS06540-RS06500) were found only in EC1 strains isolated from rice. The luminmide biosynthetic gene (FGI04_3605) was present only in the A5410 strain isolated from pineapple.

#### Prophage and Phage-Like Elements Within the *D. zeae* Complex

The Phaster analysis suggested the presence of intact prophages and prophage-like elements in five complete genomes of *D. zeae.* In total, 8 seemingly complete (intact) and 13 putatively defective (incomplete and questionable) prophage-like elements were found (Supplementary Table 5). Putatively defective (incomplete and questionable) prophage-like elements were present with a size range of 4.9–51.4 kb. Intact prophage regions were found in all five *D. zeae* strains (Supplementary Table 5); the sizes of intact prophage genomes varied from 25.8 to 46.9 kb. The genomes of EC1, Ech586, and A5410 harbored two regions of intact prophage—the first intact region included 31, 33, and 46 proteins, whereas the second intact region included 54, 33, and 53 proteins, respectively. The MS2 and PL65 genomes harbored only one region of intact prophage, 32 and 52 proteins, respectively (Supplementary Table 5).

#### Phenotypic Comparison for *D. zeae* Strains

**FIGURE 10:**
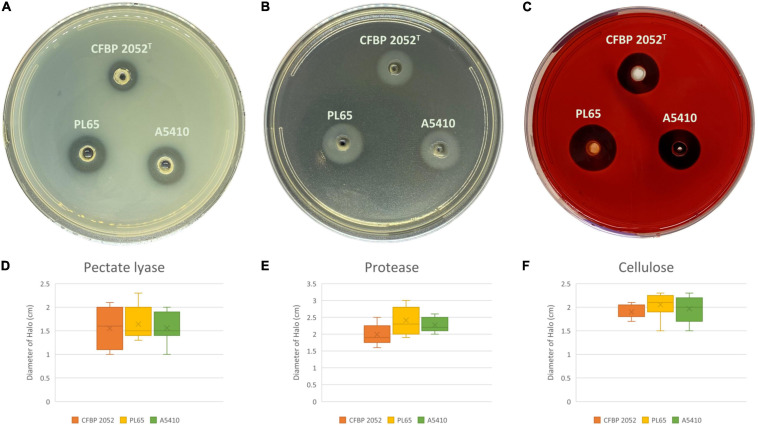
Extracellular cell wall-degrading enzymes produced by three *D. zeae* strains. The three *D. zeae* strains assayed on the **(A)** pectate lyase (Pel), **(B)** protease (Prt), and **(C)** cellulose (Cel) plates. Samples of 50 μl of overnight culture were added to the assay plate wells (3 mm in diameter) and incubated at 28°C. The Pel assay plates were treated with 4 N HCl after 10 h. The Cel assay plates were stained with 0.1% (w/v) Congo red for 10 h and decolored with 5 M NaCl. The Prt assay plates were observed after 24 h without any further treatment. **(D–F)** indicate the production of Pel, Prt, and Cel from *D. zeae* strains, respectively.

Three *D. zeae* strains [A5410, A5422 (NCPPB 2538 = CFBP 2052), and PL65], compared in phenotypic assays, showed no differences in growth rate (Supplementary Figure 8). Strains A5410, A5422, and PL65 genomes harbored the genes for Pels (*Pel* cluster), cellulases (*cel5Z*, *celH*, and *celY*), and Prts (*prt* cluster). The three strains produced Prts, Pels, and cellulases in the plate assays (Figure 10). No significant differences were detected (*p* > 0.05) among the three strains in Prt, Pel, or cellulase activity (Figure 10).

**FIGURE 11:**
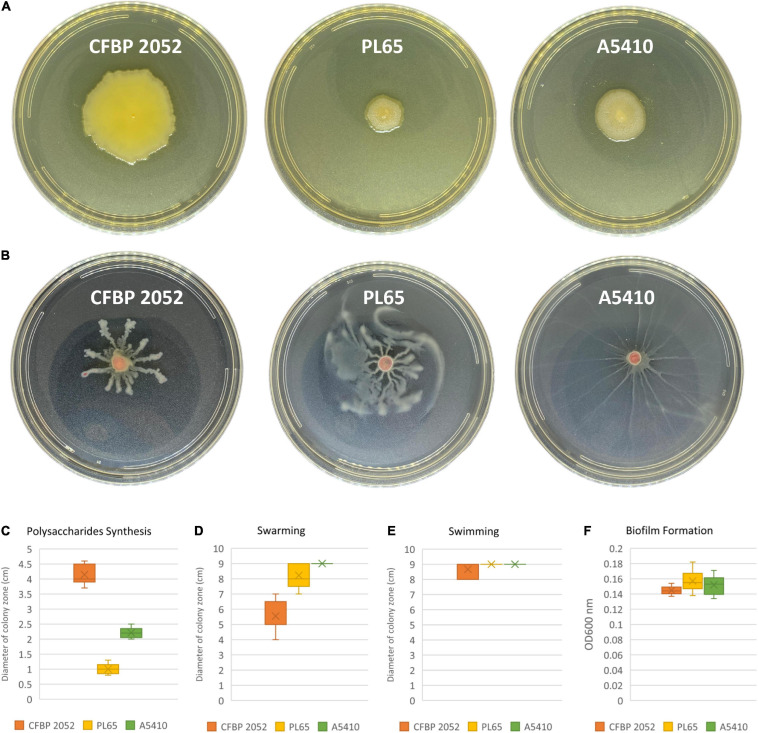
Exopolysaccharide production and motility of three *D. zeae* strains. **(A)** Exopolysaccharide [extracellular polysaccharide (EPS)] production of cells grown in Super Optimal Broth (SOB) medium of three *D. zeae* strains, and the colony diameter measured 24 h later; **(B)** swarming capacity of three *D. zeae* strains was observed in semi-solid medium, after 24 h at 28°C; **(C)** production of exopolysaccharides; **(D)** capability of swarming; **(E)** capability of swimming; and **(F)** ability of biofilm formation.

ECA, CPS, LPS, and EPS clusters were highly conserved in the three genomes. Moreover, the flagellar biosynthesis and chemotaxis proteins and type IV pilus biogenesis proteins were found in all three genomes. Biofilm formation, polysaccharide production, and motility assays were performed. All selected strains produced biofilms and there were no statistically significant differences for biofilm formation among the three strains (*p* > 0.05). The strain CFBP 2052 generated the highest exopolysaccharide on solid SOBG, whereas the strain PL65 showed the smallest colony formation on solid SOBG. PL65 and A5410 generated similar swimming and swarming zones, which were larger in comparison to the zones produced by CFBP 2052 (Figure 11). The results of EPS production assay and motility assays showed a statistically significant difference with *p* < 0.01.

### Pathogenicity Assays

**FIGURE 12:**
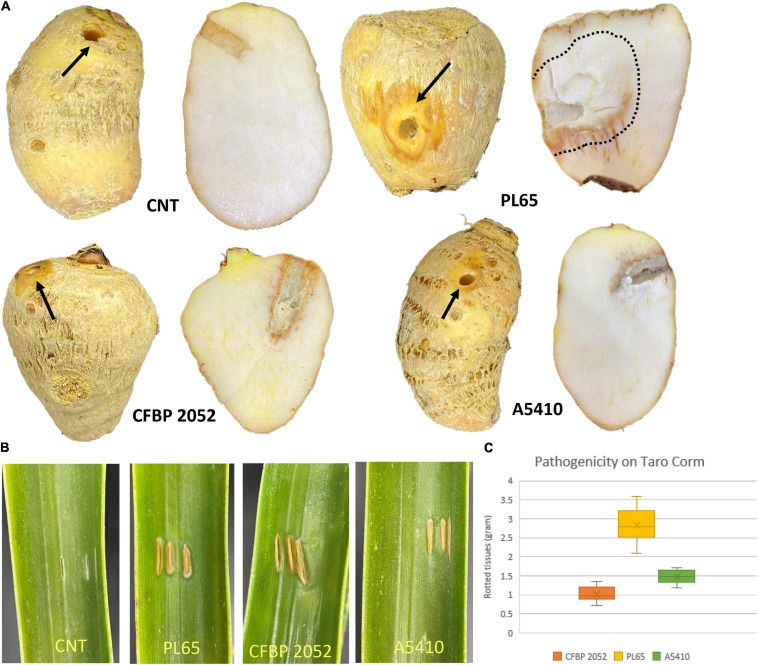
Pathogenicity of *D. zeae* strains on taro corm and pineapple leaf. **(A)** Infected and control taro corm (CNT) after incubation. Decayed tissue is indicated by black arrows and black dotted lines on the taro corm. **(B)** Infected and control leaves (CNT) 72 h after inoculation. **(C)** Percentage of macerated tissue from taro corms.

The results of pathogenicity tests on taro corms and pineapple leaves confirmed that the strains PL65 (from taro), A5410 (from pineapple), and A5422 (from maize) all infected taro and pineapple (Figure 12) and macerated tissues of both hosts. The strain PL65 developed symptoms on taro and pineapple within 6 h after inoculation—more rapidly than the other two. The taro corm pathogenicity assay showed a statistically significant difference (*p* < 0.01) in the amount of tissue macerated and PL65 was the most virulent strain (Figure 12).

## Discussion

*Dickeya zeae* strains represent a diverse and complex group within the genus *Dickeya* (Marrero et al., 2013), and recently the taxonomic position of several groups of strains within *Dickeya* has been changed. A novel strain of *D. zeae* was isolated from rice showing distinct characteristics Wang et al. (2020) and Zhang et al. (2020) separated several *D. zeae* strains (ZYY5, EC1, ZJU1202, DZ2Q, NCPPB 3531, and CSL RW192) from *D. zeae* and reclassified them as *D. oryzae*. Based on our analysis, we conclude that the two novel strains from taro and pineapple analyzed in the current research are diverse and close to the species threshold of *D. oryzae*.

The DNA structural analysis of the genomes revealed striking differences in the strains A5410 and PL65 with respect to other *D. zeae* strains. Notably, 19 and 17 genomic regions with high values of intrinsic curvature, stacking energy, and position reference were identified exclusively in the strains A5410 and PL65, respectively. Genes located in these regions are believed to be highly expressed and controlled by histone-like proteins (Vesth et al., 2013). In *Pseudomonas putida*, genomic zones displaying a high intrinsic curvature and stacking energy were associated with high recombination rates (Wu et al., 2011), leading to the prediction that the abovementioned areas detected in A5410 and PL65 might be hotspots playing significant roles during transcription and recombination processes, and hence could be essential for the survival of these strains. DNA curvature is involved in vital cell functions such as replication, transcription, recombination, and nucleosome positioning (Kozobay-Avraham, 2006). Therefore, the high intrinsic DNA curvature regions found solely in A5410 and PL65 might constitute the markers linked to the evolution of these strains into separate populations.

The key virulence factors of soft rot bacteria are their extracellular enzymes (Hugouvieux-Cotte-Pattat et al., 1996). PCWDEs (pectinases, cellulases, and proteinases) are considered as the essential virulence factors for host colonization and disease development (Davidsson et al., 2013; Charkowski et al., 2014). Pectinase enzymes such as Pels, Pem, and Pnls have been studied within *D. dadantii* 3937 (Hugouvieux-Cotte-Pattat et al., 1996). Pectinase enzymes play a significant role in the virulence and tissue maceration (Hugouvieux-Cotte-Pattat et al., 1996). Previous comparative genomic analyses revealed that the genes related to the production of PCWDEs include multiple Pels (*pelABCDEILNWXZ* and *pel10*), Pnls (*pnlGH*), Pehs (*pehKNVWX*), Pmes (*pemAB*), pectin acetyl esterases (*pae*XY), feruloyl esterases (*faeDT*), *rhi*s (*rhiEF*), and one periplasmic Gan (*GanA*) exist in various *Dickeya* species and are highly conserved (Zhou et al., 2015; Duprey et al., 2019)—our analyses demonstrated concordant results.

Gram-negative bacteria have evolved several complex secretion systems to translocate a wide range of extracellular enzymes and effector proteins from the periplasm across the outer membrane (Costa et al., 2015). The structural and mechanistic features of the types I–VI were described in the Gram-negative bacteria (Costa et al., 2015). The Prts, which are crucial for virulence, are secreted by the T1SS known as the *prtDEF* operon (Toth et al., 2006; Charkowski et al., 2012). Zhou et al. (2015) reported that *D. paradisiaca* Ech703 did not harbor the *prtDEF* genes among the *Dickeya* species, a conclusion concordant with our findings. We found that the T1SS cluster was present in all strains (EC1, MS2, Ech586, A5410, and PL65) (Supplementary Figure 4A). Previous studies have revealed that many Gram-negative bacteria use the T2SS to translocate extracellular proteins such as pectinases and cellulases (Filloux, 2004; Korotkov et al., 2012). T2SS gene cluster (*outSBCDEFGHIJKLMO)* is well-conserved among *Dickeya* species (Zhou et al., 2015; Duprey et al., 2019)—similar results were obtained in our analyses with all five strains isolated from diverse hosts including rice, banana, pineapple, taro, and philodendron (Supplementary Figure 4B). The T3SS plays a vital role in modulating plant defense for several plant bacterial pathogens, including *Pseudomonas syringae*, *Erwinia* sp., and *Xanthomonas* sp. (Deslandes and Rivas, 2012). However, recent studies indicated that a few *Pectobacterium* species such as *P. parmentieri, P. wasabiae*, and *D. paradisiaca* lack the T3SS cluster (Kim et al., 2009; Nykyri et al., 2012; Arizala and Arif, 2019; Duprey et al., 2019), and stated that T3SS is not necessary for disease development in *Pectobacterium* species (Arizala and Arif, 2019). We found that the T3SS cluster was present in all strains—EC1, MS2, Ech586, and A5410—except PL65 isolated from taro (Figure 9). The role of the *virB* (T4SS) operon was demonstrated in *P. atrosepticum* as a virulence factor (Bell et al., 2004). *D. dadantii* 3937 and *D. fangzhongdai* (ND14b, M074, and M005) encodes both types of T4SS, a *virD2/virD4/trb* locus and *virB* operon (Pédron et al., 2014). *D. zeae* complex species possessed only one type of T4SS, *virB* operon (Supplementary Figure 5). Interestingly, PL65 and MS2 harbored *virB1* and *virB2* (Supplementary Figure 5). Previous studies indicated that *vir*B1 forms a borehole in the peptidoglycan layer that enables a complex T4SS assembly to occur, and the proteins *VirB2* and *VirB5* constitute T4SS extracellular pilus (Chandran et al., 2009; Fronzes et al., 2009). T5SS, a two-partner secretion system, consists of an outer membrane protein and a hemagglutinin repeat region. The T5SSs are encoded within the T3SS gene cluster in some *Dickeya* species (Rojas et al., 2002). Our analysis showed that *D. zeae* genomes harbor the T5SS, but might not be functional because some of its components are encoded by pseudogenes. In 2006, T6SS was recognized as a distinct class of bacterial protein secretion system (Mougous et al., 2006) and identified as a virulence locus in *Pseudomonas aeruginosa, Burkholderia* species, and *Salmonella enterica* (Mougous et al., 2006; Hood et al., 2010; Jani and Cotter, 2010; Schwarz et al., 2010; Bernal et al., 2018). Previous analysis indicated that *D. zeae* and *D. chrysanthemi* have an identical T6SS locus. However, the biological function of T6SS in *Dickeya* has not yet been determined (Sarris et al., 2012; Zhou et al., 2015). In this study, a gene cluster encoding T6SS was found in all strains (EC1, MS2, Ech586, A5410, and PL65) (Supplementary Figure 6B).

Flagellar biosynthesis and chemotaxis proteins were found in all five strains (EC1, Ech586, MS2, A5410, and PL65). Previously, Zhou et al. (2015) have proven that EC1, DZZ2Q, and ZJU1202 strains isolated from rice possessed the flagellar biosynthesis gene clusters. In many plant pathogenic bacteria, flagellar proteins are responsible for cell motility and secretion and vesicular transport (Jahn et al., 2008), and motility lends to virulence (Panopoulos and Schroth, 1974; Mulholland et al., 1993; Chesnokova et al., 1997; Tans-Kersten et al., 2001). Flagella are used for both swimming and swarming motility (Yi et al., 2000). Individual swimming cells perceive a chemical signal *via* methyl-accepting chemotaxis proteins responsible for cell motility and signal transduction (Yi et al., 2000). It has been demonstrated that mutation within chemotactic genes (*cheW, cheB, cheY*, and *cheZ*) caused a substantial reduction in swimming motility (Antúnez-Lamas et al., 2009). Strains PL65 and A5410 isolated from taro and pineapple, respectively, were similar with respect to swimming motility, but PL65 was the most virulent on taro corms among the three strains tested (Figures 11, 12). Jahn et al. (2008) have proven that the mutation of the *fliA* gene encoding a sigma factor obstructed the bacterial motility and limited Pels production and bacterial attachment to plant tissues in *D. dadantii* 3937. These results show that flagellar biosynthesis and chemotaxis proteins are associated with virulence. Another virulence factor studied in plant pathogenic bacteria, such as *Ralstonia* and *Xylella*, is the type IV pilus (Burdman et al., 2011). The type IV pilus assembly encoded by *pil* genes is responsible for twitching motility in *P. aeruginosa* and *D. aquatica* (Maier and Wong, 2015; Duprey et al., 2019). We found this *pil* gene cluster in all *D. zeae* strains.

*Dickeya* species produce secondary metabolites such as thiopeptide, cyanobactin, zeamine, and oocydin (Zhou J. et al., 2011; Zhou et al., 2015; Alič et al., 2019; Duprey et al., 2019). The polyketides (PKS) and non-ribosomal peptides (NRPS) are the two representative classes of enzymes that synthesize essential secondary metabolites (Blin et al., 2013). The zeamine gene cluster is well known among the secondary bioactive metabolites for *Dickeya* species, such as *D. fangzhongdai, D. solani*, and the *D. oryzae* strains previously classified as *D. zeae* (Zhang et al., 2013; Zhou et al., 2015; Alič et al., 2019; Duprey et al., 2019). *Dickeya oryzae* strains (ZJU1202, DZ2Q, EC1, and ZYY5) isolated from rice possessed the zeamine (*zms*) gene cluster (Zhou et al., 2015; Wang et al., 2020). The zeamine cluster is capable of inhibiting rice seed germination and growth (Zhou J. et al., 2011; Zhou et al., 2015). This distinction from strains belonging to *D. zeae* was used in describing a novel species of *Dickeya* (Wang et al., 2020). Additionally, we observed that strain EC1 produced the antifungal compound oocydin *via* non-ribosomal peptide synthases (NRPs) and polyketide synthases (PKs). Oocydin is responsible for its strong antimicrobial activity against plant pathogenic fungi and oomycetes (Matilla et al., 2015). Similar cluster sequences were also present in other *Dickeya* species, namely *D. solani*, *D. dianthicola*, *D. zeae*, *D. chrysanthemi*, *D. fangzhongdai*, and *D. paradisiaca* (Alič et al., 2019; Duprey et al., 2019). The antioxidant indigoidine is a well-known secondary metabolite, produced by all *Dickeya* (Nasser et al., 2013). We confirmed that the *D. zeae* strains harbored the antioxidant indigoidine.

The synthesis of clusters of different types of polysaccharides, such as CPS, EPS, and lipo-oligo/polysaccharide, are considered as the important virulence factors that enable bacteria to bind to the host cell surface (Kuhn et al., 1988; Reeves et al., 1996; Roberts, 1996; Toth et al., 2003, 2006; Bell et al., 2004; Nykyri et al., 2012; Arizala and Arif, 2019). EPS is a main component of the bacterial biofilm matrix and is responsible for adhesion to plant surfaces (Flemming and Wingender, 2010). Strains in the genus *Pectobacterium* harbored an EPS biosynthetic cluster (*wza-wzb-wzc-wzx*) (Arizala and Arif, 2019). In this study, we found that all five *D. zeae* strains harbored the EPS cluster, and we observed no differences in biofilm formation among these strains. The *cps* (CPS) cluster was not observed in some *Pectobacterium* strains (Arizala and Arif, 2019). We determined that the strain EC1 possessed the LOP- and EPS-gene cluster; however, the *cps* cluster was absent. Moreover, in the solid SOBG medium assay, strain EC1 produced the greatest amount of polysaccharides. Additional factors are involved in biofilm formation such as swimming, swarming, and twitching motility.

In this study, additional important genes were identified and predicted to play functional roles. These genes were annotated and associated with the production of antimicrobials, nitrogen fixation, and the uptake and catabolism of aromatic compounds. We demonstrated that only the strain A5410 isolated from pineapple harbored the nitrogen fixation cluster (*nifABCDEHKLMNSTUVQWXYZ*), the *arsC* (arsenic resistant gene) gene, and the carboxymuconolactone decarboxylase gene. The nitrogen fixation cluster was also present in *D. solani* and *P. atrosepticum* (Bell et al., 2004; Golanowska, 2015). Carboxymuconolactone decarboxylase participates in the catalysis of aromatic compounds to produce acetyl- or succinyl-CoA in prokaryotes and yeast (Cheng et al., 2017). Carboxymuconolactone decarboxylase was present in *Azotobacter vinelandii, Acinetobacter calcoaceticus*, and *Pseudomonas putida* (Yeh et al., 1981). Bacterial survival within a specific environment is linked to the ability of bacteria to cope with toxic compounds. The acquisition of arsenic clusters (*ars*) confers the ability of bacteria to resist high concentrations of inorganic arsenic present in the environment (Fekih et al., 2018). A recent study showed that *P. atrosepticum, P. brasiliense*, *P. peruviense*, and *P. versatile* (formerly proposed as *Candidatus Pectobacterium* maceratum) possess four genes in arsenic clusters: *arsC*, *arsB*, *arsR*, and *arsH* (Bell et al., 2004; Arizala and Arif, 2019). The *arsC* and *arsH* genes in *Pectobacterium* are vital for the survival in an arsenic-rich environment (Arizala and Arif, 2019).

The CRISPRs and the Cas (CRISPR-Cas) is widely distributed and found in at least half of the bacteria and in almost all archaea (Haft et al., 2005). The CRISPR-Cas is defined as the immune system that can protect against bacteriophages and foreign plasmids (Mojica et al., 2005; Makarova et al., 2011; Rath et al., 2015). Three types of CRISPR-Cas systems (types I–III) were classified based on Cas3, Cas9, and Cas10 proteins. The type I comprises further subtypes (e.g., I-A to I-F), each is characterized by a specific set of proteins (Przybilski et al., 2011). Two types of CRISPR-Cas systems (cas-type III and cas-subtype IE/IF) were described among the strains of *D. zeae.* Previously, the cas-subtype IE was observed in *D. fangzhongdai, D. dadantii*, *D. zeae*, and *D. paradisiaca*, and the cas-subtype IC was identified in *D. dadantii* and *D. dianthicola* (McGhee and Sundin, 2012; Pédron et al., 2014; Medina-Aparicio et al., 2018; Zhang et al., 2018). In our analyses, orphan CRISPRs were found throughout the four analyzed *Dickeya* genomes (Supplementary Table 3). Some orphan CRISPRs appeared to exert their function far away from the *cas* locus, although they might not be functional (Zhang and Ye, 2017). Similarly, orphan CRISPRs were also found in some *Pectobacterium* species, and these might be functional or just remnants of previous CRISPR-Cas systems (Arizala and Arif, 2019).

## Conclusion

We present two high-quality complete genome sequences of novel *D. zeae* strains PL65 and A5410 isolated from taro and pineapple in Hawaii. Detailed comparative genomic analyses were performed by using the selected strains along with the three other strains retrieved from the NCBI GenBank genome database. For taxonomic and phylogenomic analyses, representative strains from other species were also included. Several groups of virulence genes, such as those coding for cell wall-degrading extracellular enzymes, T1SS gene cluster *prtDEF*, T2SS gene cluster (*out* gene cluster), T5SS gene cluster, T6SS gene cluster, flagellar and chemotaxis gene clusters, certain polysaccharide synthesis clusters, and the type IV pilus gene cluster, are highly or fully conserved in all five genomes isolated from the different hosts. Interestingly, T3SS and T4SS gene clusters were absent in the strain PL65 isolated from taro. We also found that the T4SS gene cluster was absent in MS2. Importantly, a range of unique genes, such as an arsenic-resistant gene and a nitrogen fixation cluster gene, associated with virulence were identified in the pineapple strain, A5410. Intriguingly, the zeamine (*zms*) gene cluster and oocydin gene cluster were found only in strain EC1, which was isolated from rice.

## Data Availability Statement

The datasets presented in this study can be found in online repositories. The names of the repository/repositories and accession number(s) can be found below: CP040817: https://www.ncbi.nlm.nih.gov/nuccore/CP040817 and CP040816: https://www.ncbi.nlm.nih.gov/nuccore/CP040816.

## Author Contributions

MA conceived and designed the study. GB performed the experiments and wrote the manuscript. GB and DA performed the analyses. MA, SD, JZ, JH, and AA revised the manuscript and provided ideas and support for the final submission. All authors reviewed the manuscript.

## Author Disclaimer

The content is solely the responsibility of the authors and does not necessarily represent the official views of the funding agencies.

## Conflict of Interest

The authors declare that the research was conducted in the absence of any commercial or financial relationships that could be construed as a potential conflict of interest.

## Publisher’s Note

All claims expressed in this article are solely those of the authors and do not necessarily represent those of their affiliated organizations, or those of the publisher, the editors and the reviewers. Any product that may be evaluated in this article, or claim that may be made by its manufacturer, is not guaranteed or endorsed by the publisher.

## Footnotes

1
http://ggdc.dsmz.de/ggdc.php#

2
https://www.displayr.com/

3
http://phaster.ca/

## Supplementary Material

Supplementary Figure 1 Pairwise heatmap based on the average nucleotide identity (ANI) and digital DNA–DNA hybridization (dDDH) values of 14 *Dickeya* and a *Pectobacterium* species. The upper diagonal displays ANI data whereas the lower diagonal depicts the *in silico* dDDH values. Cut-off values for species delineation are 95–96% and 70% for ANI and dDDH, respectively.

Supplementary Figure 2 The phylogenetic tree of *Dickeya* and *Pectobacterium* species by using dDDH and ANI data. **(A)** The dDDH phylogenetic tree was inferred with FastME 2.1.6.1 from TYGS_GBDP distances calculated from genome sequences. **(B)** The ANI phylogenetic tree was generated for the *Dickeya* species strains based on whole-genome alignment using the neighbor-joining method. The Jukes–Cantor model was used for analysis with 1,000 bootstraps.

Supplementary Figure 3 Amino acid and codon usage for all 14 genomes calculated based on the genes identified. The percentage of **(A)** codon and **(B)** amino acid usage were plotted in two heatmaps using R. Organisms were marked 1–14; *Dickeya zeae* 1, PL65; 2, A5410; 3, MS2; 4, Ech586; 5, EC1; 6, *D. undicola* FVG10-MFV-A16; 7, *D. aquatica* 174/2; 8, *D. lacustris* S29; 9, *D. solani* IPO 2222; 10, *D. dadantii* 3937; 11, *D. dianthicola* ME23; 12, *D. fangzhongdai* PA1; 13, *D. paradisiaca* Ech703; and 14, *D. chrysanthemi* Ech1591.

Supplementary Figure 4 Comparison of the genetic organization of **(A)** type I secretion system (T1SS) and **(B)** type II secretion system (T2SS) among five *D. zeae* strains. The arrow position represented forward/reverse gene orientation. Arrow color signified specific gene composition within the T1SS and T2SS. Gene names were provided at the top and bottom of the linear graph. A pairwise alignment between the linear sequences was rendered based upon Basic Local Alignment Search Tool (BLAST) algorithm with cut-off values from 66 to 100% and 82 to 100%, T1SS and T2SS, respectively. Regions with higher nucleotide identity were displayed with shaded gray.

Supplementary Figure 5 Comparison of the genetic organization of type IV secretion system (T4SS) among five *D. zeae* strains. The arrow position represented a forward/reverse gene orientation. Arrow color signified specific gene composition within the T4SS. A pairwise alignment between the linear sequences was rendered based upon the BLAST algorithm with cut-off values from 91 to 100%. Regions with a higher nucleotide identity were displayed with a shaded gray.

Supplementary Figure 6 Comparison of the genetic organization of **(A)** type V secretion system (T5SS) and **(B)** type VI secretion system (T6SS) among five *D. zeae* strains. The arrow position represented forward/reverse gene orientation. Arrow color signified specific gene composition within the T5SS and T6SS. Gene names were provided at the top and bottom of the linear graph. A pairwise alignment between the linear sequences was rendered based upon BLAST algorithm with cut-off values from 68 to 100%. Regions with higher nucleotide identity were displayed with a shaded gray.

Supplementary Figure 7 Diagram of the clustered regularly interspaced short palindromic repeats (CRISPR) with CRISPR associated proteins (*Cas*) system in five *D. zeae* strains. **(A)** The subtype I-E Cas, **(B)** The subtype I-F Cas, and **(C)** The type III-A Cas. Orange arrows represent CRISPR repeats.

Supplementary Figure 8 Bacterial growth curve of *D. zeae* strains (CFBP 2052^*T*^, A5410, and PL65). Bacterial cultures were grown with Nutrient Broth at 37°C with continuous shaking. These data represent three separate experiments.

Supplementary Table 1 About 86 virulence-related genes include cell wall-degrading enzyme genes, flagellar and chemotaxis genes, twitching motility genes, polysaccharide biosynthesize gene, clusters from *Dickeya* species, and their locus tag within each genome.

Supplementary Table 2 General genome characteristics described for the five complete genomes of *Dickeya zeae.*

Supplementary Table 3 A detailed description of the main features of clustered regularly interspaced short palindromic repeats (CRISPRs) found in *D. zeae* strains.

Supplementary Table 4 Identification of the secondary metabolite gene clusters using AntiSMASH in five genomes of *Dickeya* species.

Supplementary Table 5 Prophages identified in *D. zeae* strains using Phaster.
