# Supplementary figures and images for: Genomic and Phenotypic Biology of Novel Strains of Dickeya zeae Isolated From Pineapple and Taro in Hawaii: Insights Into Genome Plasticity, Pathogenicity, and Virulence Determinants
Source: Front Plant Sci. 2021 Aug 11;12:663851. doi: 10.3389/fpls.2021.663851 (PMC8386352; doi:10.3389/fpls.2021.663851)

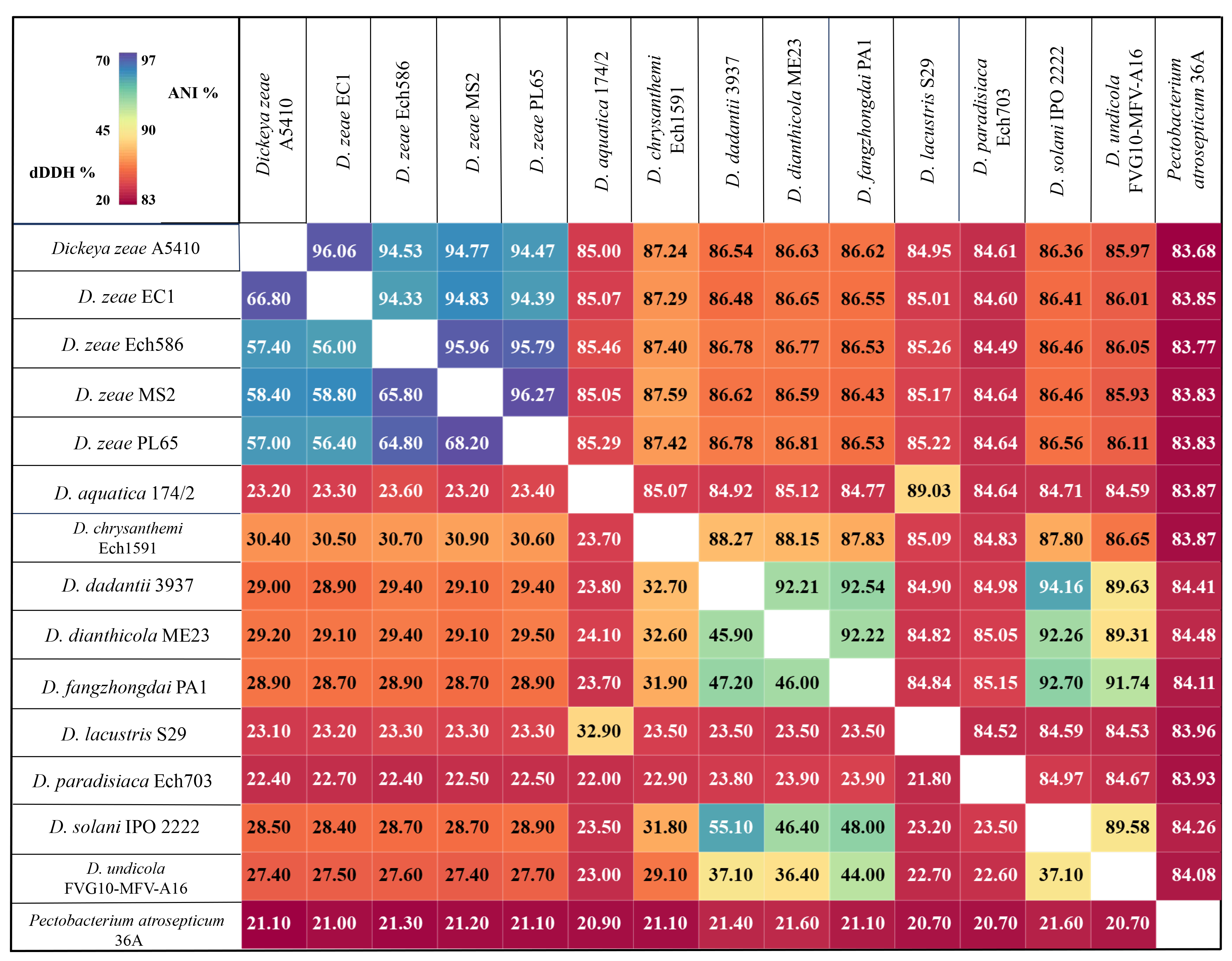

Supplement: Supplementary Figure 1 — Pairwise heatmap based on the average nucleotide identity (ANI) and digital DNA–DNA hybridization (dDDH) values of 14 Dickeya and a Pectobacterium species. The upper diagonal displays ANI data whereas the lower diagonal depicts the in silico dDDH values. Cut-off values for species delineation are 95–96% and 70% for ANI and dDDH, respectively. [file Image_1.TIF]

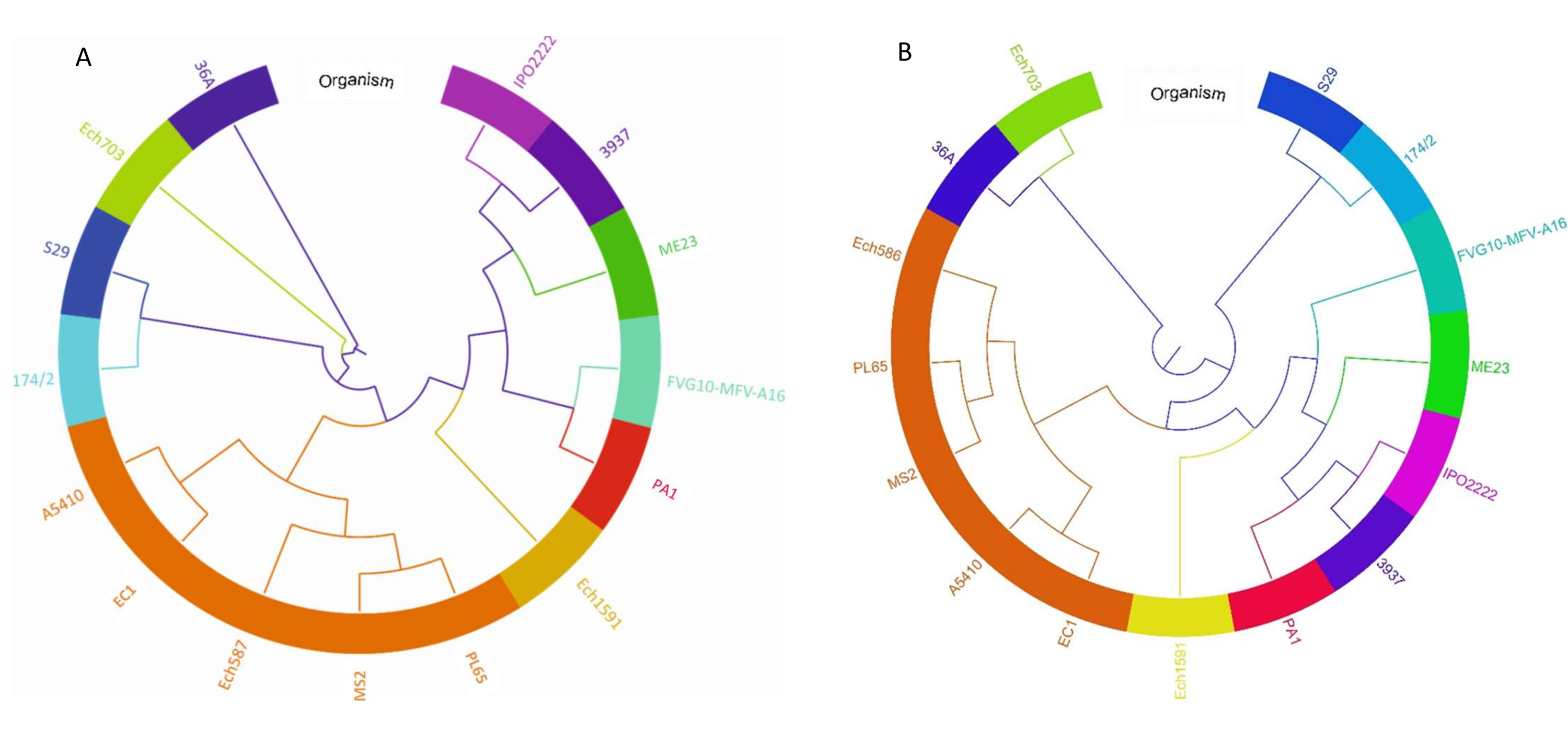

Supplement: Supplementary Figure 2 — The phylogenetic tree of Dickeya and Pectobacterium species by using dDDH and ANI data. (A) The dDDH phylogenetic tree was inferred with FastME 2.1.6.1 from TYGS_GBDP distances calculated from genome sequences. (B) The ANI phylogenetic tree was generated for the Dickeya species strains based on whole-genome alignment using the neighbor-joining method. The Jukes–Cantor model was used for analysis with 1,000 bootstraps. [file Image_2.TIF]

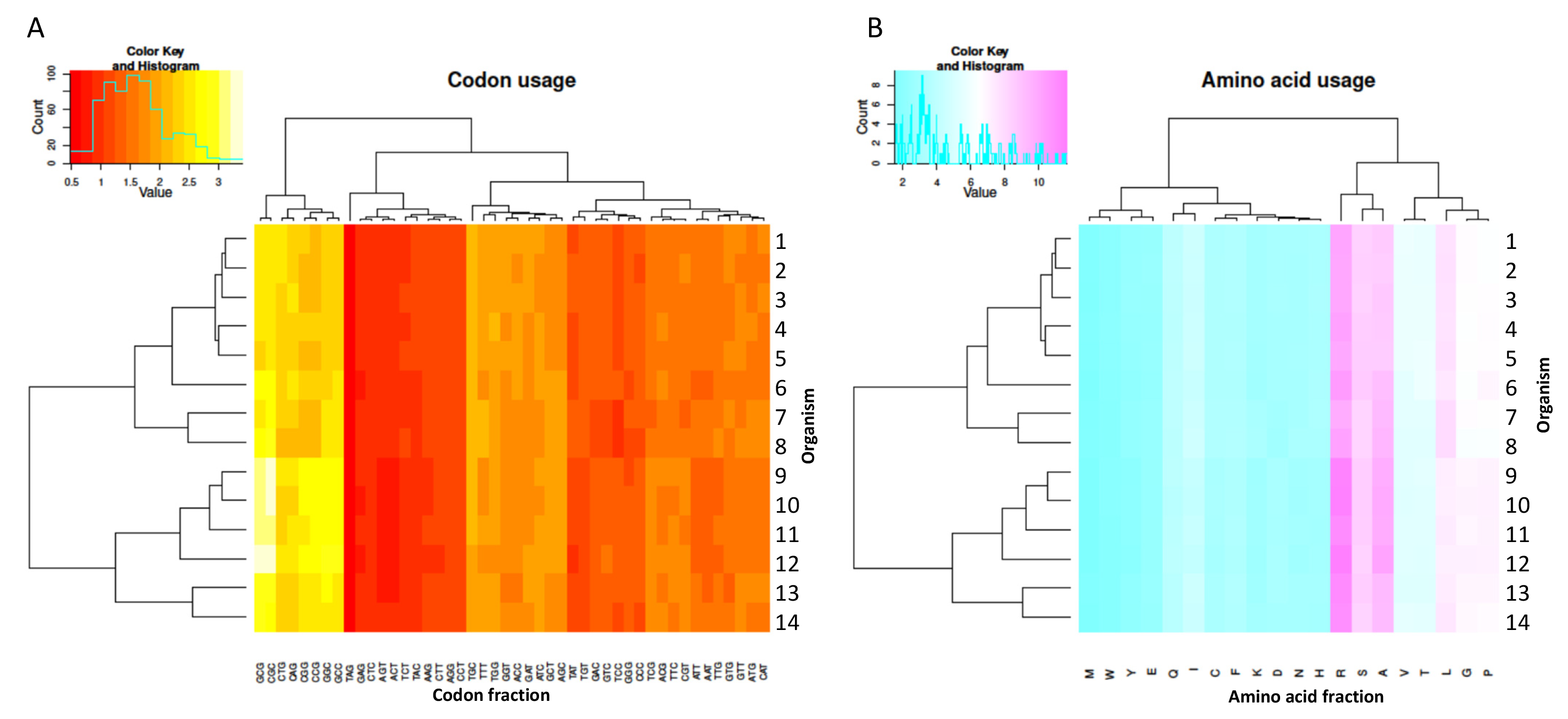

Supplement: Supplementary Figure 3 — Amino acid and codon usage for all 14 genomes calculated based on the genes identified. The percentage of (A) codon and (B) amino acid usage were plotted in two heatmaps using R. Organisms were marked 1–14; Dickeya zeae 1, PL65; 2, A5410; 3, MS2; 4, Ech586; 5, EC1; 6, D. undicola FVG10-MFV-A16; 7, D. aquatica 174/2; 8, D. lacustris S29; 9, D. solani IPO 2222; 10, D. dadantii 3937; 11, D. dianthicola ME23; 12, D. fangzhongdai PA1; 13, D. paradisiaca Ech703; and 14, D. chrysanthemi Ech1591. [file Image_3.TIF]

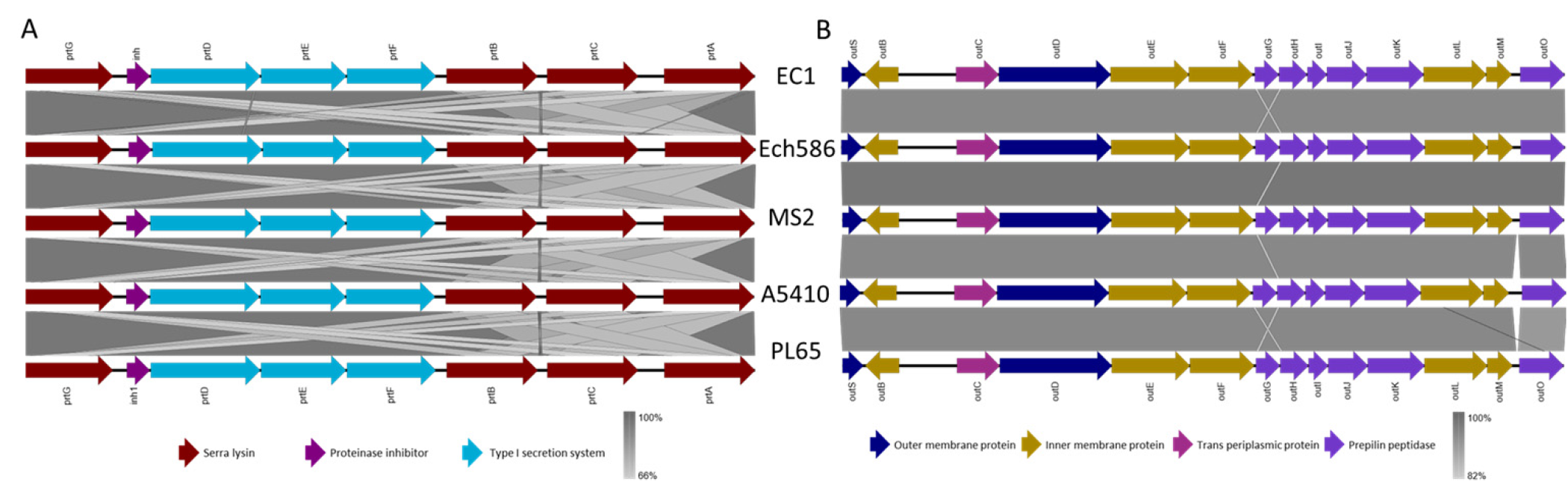

Supplement: Supplementary Figure 4 — Comparison of the genetic organization of (A) type I secretion system (T1SS) and (B) type II secretion system (T2SS) among five D. zeae strains. The arrow position represented forward/reverse gene orientation. Arrow color signified specific gene composition within the T1SS and T2SS. Gene names were provided at the top and bottom of the linear graph. A pairwise alignment between the linear sequences was rendered based upon Basic Local Alignment Search Tool (BLAST) algorithm with cut-off values from 66 to 100% and 82 to 100%, T1SS and T2SS, respectively. Regions with higher nucleotide identity were displayed with shaded gray. [file Image_4.TIF]

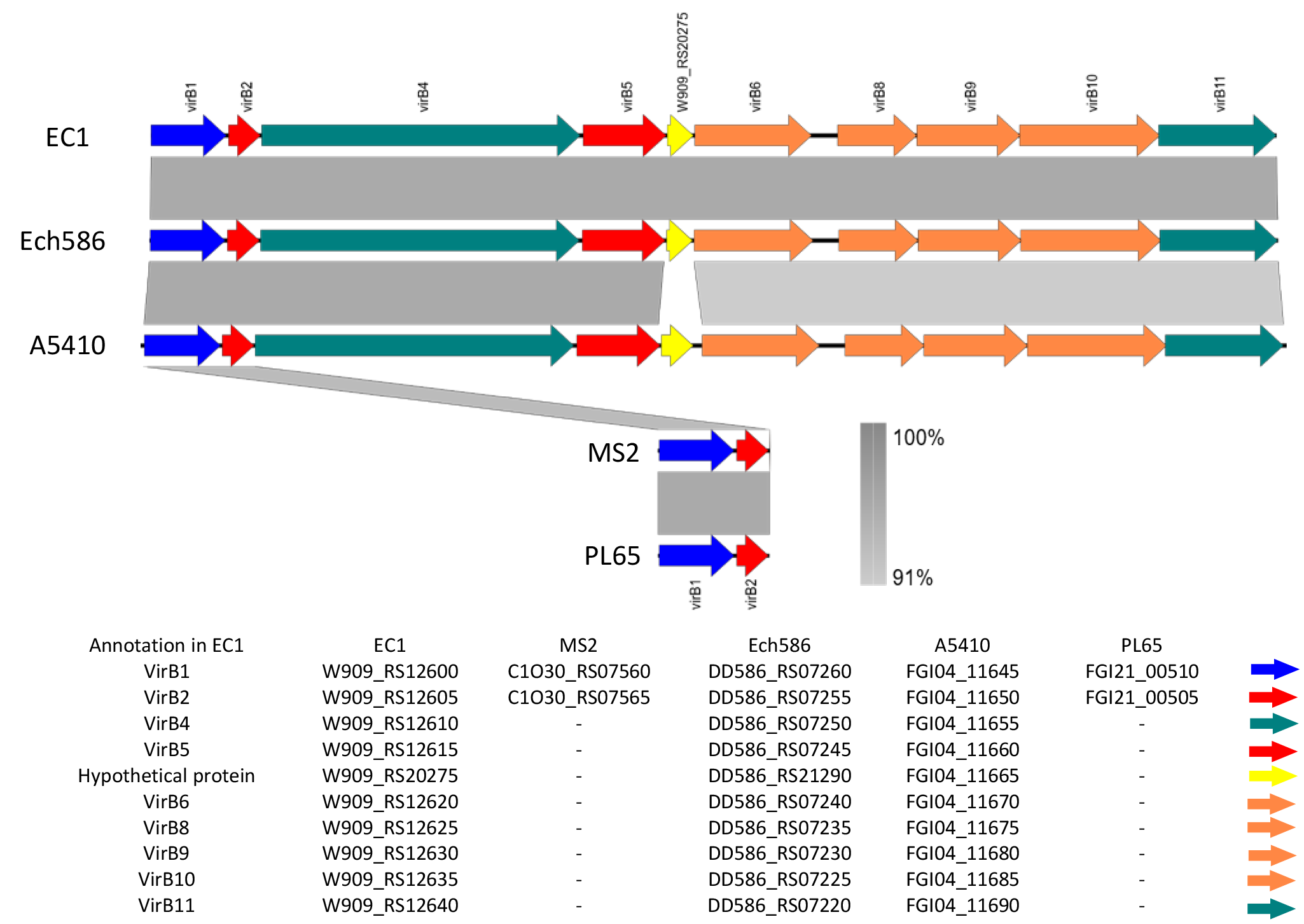

Supplement: Supplementary Figure 5 — Comparison of the genetic organization of type IV secretion system (T4SS) among five D. zeae strains. The arrow position represented a forward/reverse gene orientation. Arrow color signified specific gene composition within the T4SS. A pairwise alignment between the linear sequences was rendered based upon the BLAST algorithm with cut-off values from 91 to 100%. Regions with a higher nucleotide identity were displayed with a shaded gray. [file Image_5.TIF]

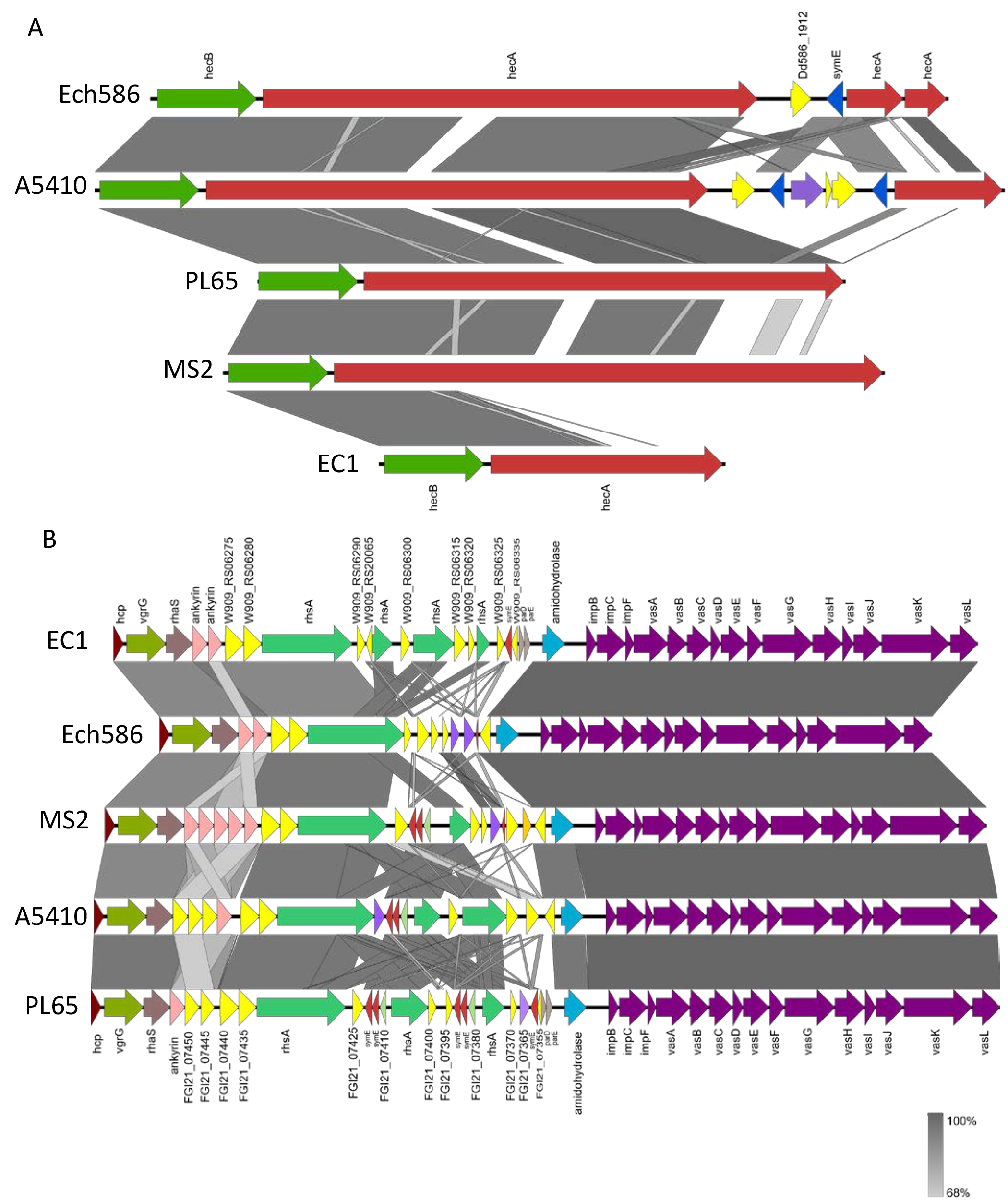

Supplement: Supplementary Figure 6 — Comparison of the genetic organization of (A) type V secretion system (T5SS) and (B) type VI secretion system (T6SS) among five D. zeae strains. The arrow position represented forward/reverse gene orientation. Arrow color signified specific gene composition within the T5SS and T6SS. Gene names were provided at the top and bottom of the linear graph. A pairwise alignment between the linear sequences was rendered based upon BLAST algorithm with cut-off values from 68 to 100%. Regions with higher nucleotide identity were displayed with a shaded gray. [file Image_6.TIF]

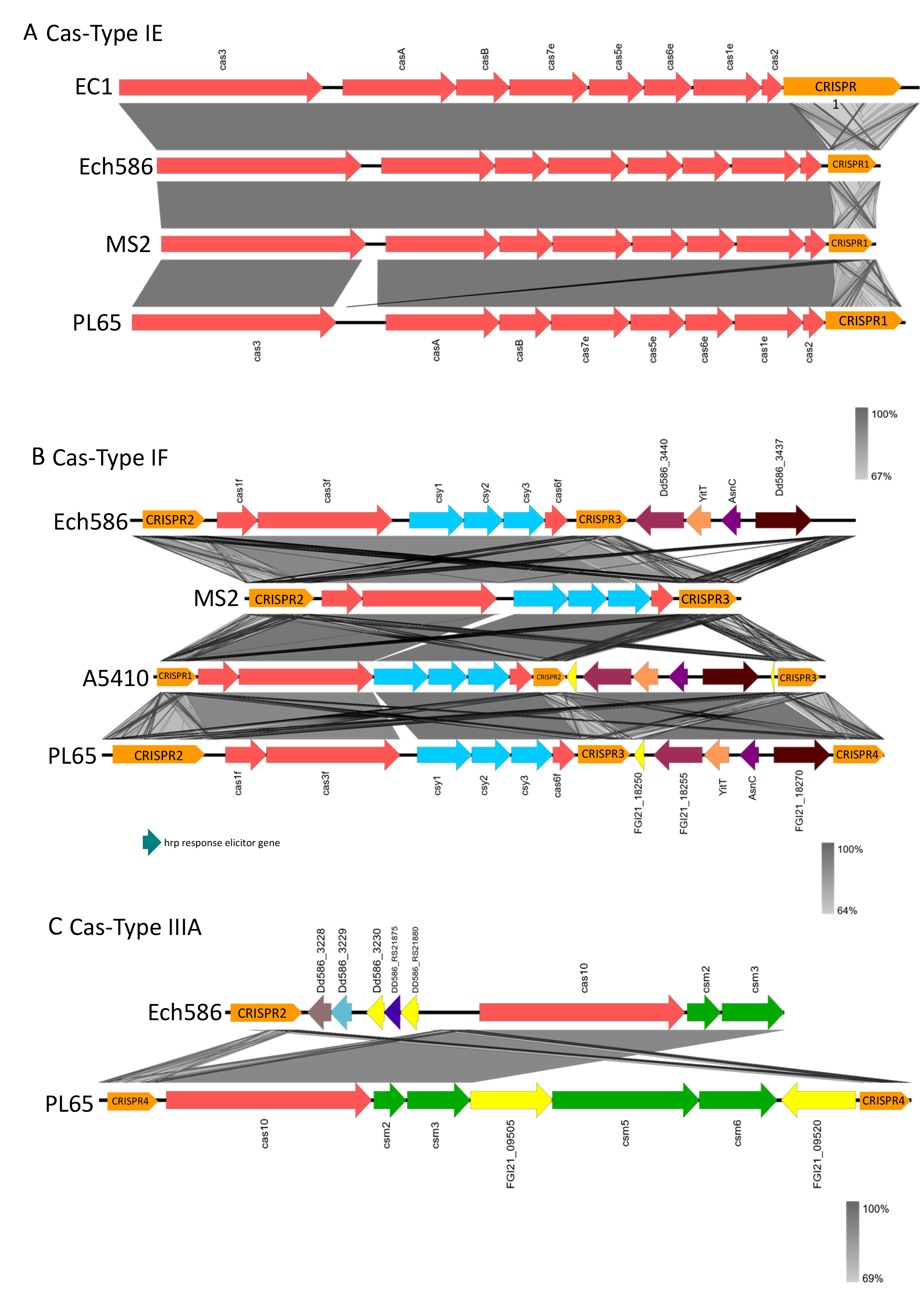

Supplement: Supplementary Figure 7 — Diagram of the clustered regularly interspaced short palindromic repeats (CRISPR) with CRISPR associated proteins (Cas) system in five D. zeae strains. (A) The subtype I-E Cas, (B) The subtype I-F Cas, and (C) The type III-A Cas. Orange arrows represent CRISPR repeats. [file Image_7.TIF]

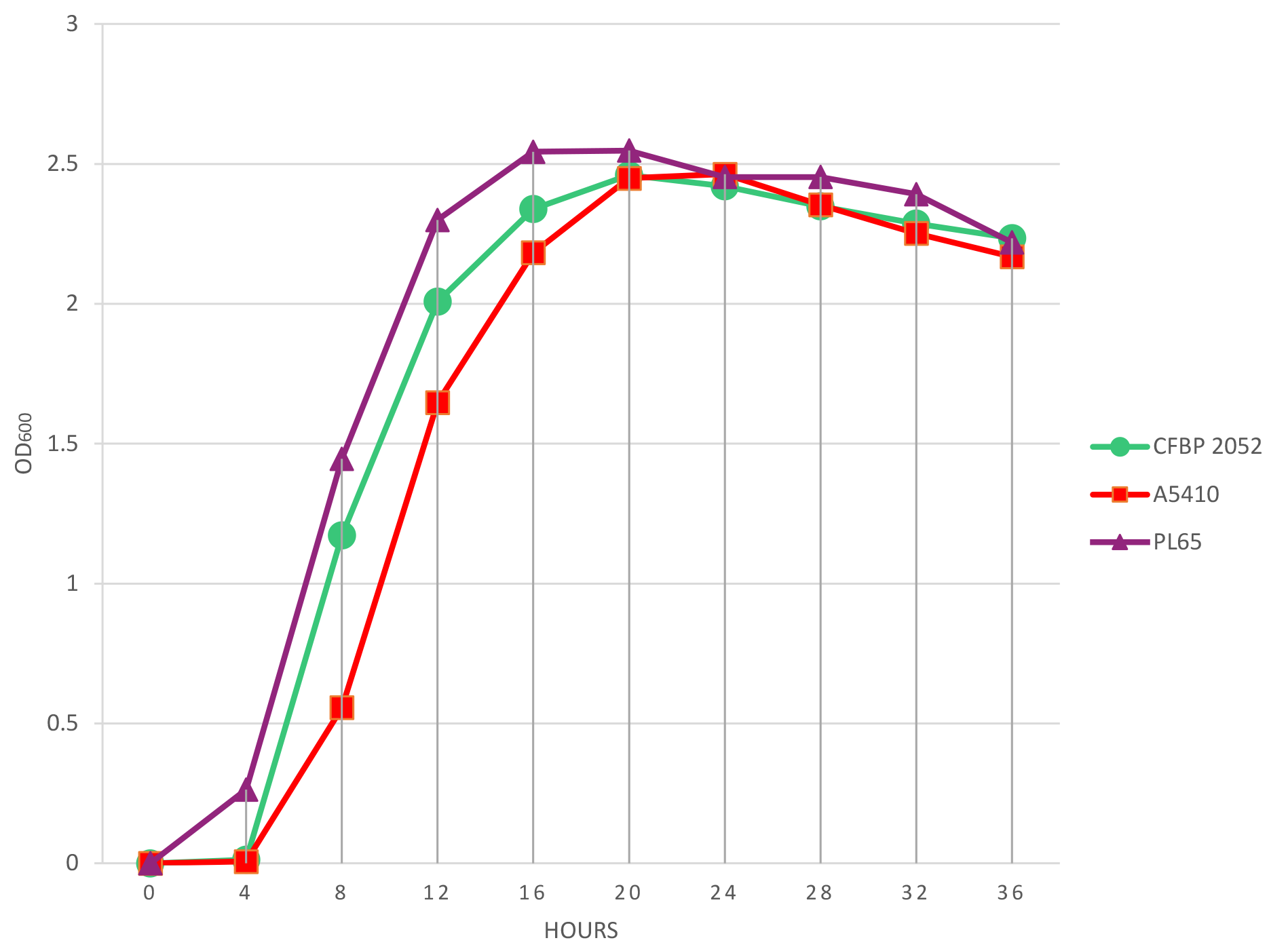

Supplement: Supplementary Figure 8 — Bacterial growth curve of D. zeae strains (CFBP 2052T, A5410, and PL65). Bacterial cultures were grown with Nutrient Broth at 37°C with continuous shaking. These data represent three separate experiments. [file Image_8.TIF]
