# Supplementary material for: Genomic and Phenotypic Biology of Novel Strains of Dickeya zeae Isolated From Pineapple and Taro in Hawaii: Insights Into Genome Plasticity, Pathogenicity, and Virulence Determinants
Source: Front Plant Sci. 2021 Aug 11;12:663851. doi: 10.3389/fpls.2021.663851 (PMC8386352; doi:10.3389/fpls.2021.663851)
Supplement: Supplementary Table 2 — General genome characteristics described for the five complete genomes of Dickeya zeae. [file Table_2.DOCX]

**Table S2.** General genome characteristics described for the five complete genomes of *Dickeya zeae* strains

| **Genome features** | **EC1** | **Ech586** | **MS2** | **A5410** | **PL65** |
| --- | --- | --- | --- | --- | --- |
| Length (bp) | 4532364 | 4818394 | 4740052 | 4779199 | 4749968 |
| % GC | 53.4 | 53.6 | 53.4 | 53.5 | 53.6 |
| CDS (coding) | 4012 | 4269 | 4221 | 4305 | 4182 |
| rRNAs (5S, 16S, 23S) | 8, 7, 7 | 8, 7, 7 | 8, 7, 7 | 8, 7, 7 | 8, 7, 7 |
| tRNAs | 88 | 76 | 75 | 75 | 75 |
| ncRNAs | 12 | 6 | 6 | 8 | 9 |
| Pseudogenes | 65 | 64 | 57 | 90 | 87 |
| Hypothetical proteins* | 760 | 565 | 924 | 879 | 873 |
| Virulence factor^*^ | 71 | 72 | 71 | 69 | 69 |
| Transporter^*^ | 176 | 180 | 186 | 183 | 183 |
| Antibiotic resistance^*^ | 37 | 37 | 42 | 42 | 42 |
| Drug target [TTD]^*^ | 27 | 27 | 26 | 27 | 27 |

GC, Guanine-Cytosine; CDS, Coding Sequence Regions; EC number, Enzyme Commission Number; TTD, Therapeutic Target Database.

*Data retrieved from the bioinformatics PATRIC webserver.
