# Supplementary material for: Genomic and Phenotypic Biology of Novel Strains of Dickeya zeae Isolated From Pineapple and Taro in Hawaii: Insights Into Genome Plasticity, Pathogenicity, and Virulence Determinants
Source: Front Plant Sci. 2021 Aug 11;12:663851. doi: 10.3389/fpls.2021.663851 (PMC8386352; doi:10.3389/fpls.2021.663851)
Supplement: Supplementary Table 3 — A detailed description of the main features of clustered regularly interspaced short palindromic repeats (CRISPRs) found in D. zeae strains. [file Table_3.DOCX]

**Table S3**. Detailed description of main features of CRISPR found in *Dickeya zeae* strains

| ***Dickeya zeae* Complex** | **CRISPR-Cas Type** | **No. of CRISPRs** | **Start Position** | **End Position** | **Strand** | **Repeats Length (bp)** | **Sp.** | **Direct Repeat Consensus** |
| --- | --- | --- | --- | --- | --- | --- | --- | --- |
| **EC1** | Cas-Type IE | CRISPR-1 | 402225 | 403962 | + | 29 | 28 | GTGTTCCCCGCGCCAGCGGGGATAAACCG |
|  | Orphan CRISPR | CRISPR-2 | 669453 | 672475 | + | 29 | 49 | GTGTTCCCCGCGCCAGCGGGGATAAACCG |
|  |  | CRISPR-3 | 3615548 | 3615877 | + | 28 | 8 | GTGAACTGCCGCATAGGCAGCTTAGAAA |
| **Ech586** | Cas-Type IE | CRISPR-1 | 406153 | 406852 | + | 29 | 11 | GTGTTCCCCGCGCCAGCGGGGATAAACCG |
|  | Cas-Type IF | CRISPR-2 | 3915410 | 3916157 | + | 28 | 12 | GTGAACTGCCGCATAGGCAGCTTAGAAA |
|  |  | CRISPR-3 | 3921209 | 3922317 | - | 28 | 18 | TTTCTAAGCTGCCTACTCGGCAGTGAAC |
|  |  | CRISPR-4 | 3931312 | 3933079 | + | 28 | 29 | GTTCACTGCCGAGTAGGCAGCTTAGAAA |
|  | Cas- Type IIIA | CRISPR-5 | 3674324 | 3674626 | + | 23 | 4 | CCCTGACTGAAGGGATTAAGACT |
|  | Orphan CRISPR | CRISPR-6 | 682808 | 683264 | + | 29 | 7 | GTGTTCCCCGCGCCAGCGGGGATAAACCG |
| **MS2** | Cas-Type IE | CRISPR-1 | 421680 | 422196 | + | 29 | 8 | GTGTTCCCCGCGCCAGCGGGGATAAACCG |
|  | Cas-Type IF | CRISPR-2 | 3823733 | 3825139 | - | 28 | 23 | TTTCTAAGCTGCCTATGCGGCAGTGAAC |
|  |  | CRISPR-3 | 3834173 | 3835400 | + | 28 | 20 | GTTCACTGCCGGATAGGCAGCTTAGAAA |
|  | Orphan CRISPR | CRISPR-4 | 677511 | 678089 | + | 29 | 9 | GTGTTCCCCGCGCCAGCGGGGATAAACCG |
| **A5410** | Cas-Type IF | CRISPR-1 | 310100 | 310668 | - | 29 | 9 | TTTCTAAGCTGCCTACTCGGCAGTGAAC |
|  |  | CRISPR-2 | 319266 | 319894 | + | 28 | 10 | GTTCACTGCCGTGTAGGCAGCTTAGAAA |
|  |  | CRISPR-3 | 325129 | 326297 | - | 29 | 19 | TTTCTAAGCTGCCTACACGGCAGTGAAC |
| **PL65** | Cas-Type IE | CRISPR-1 | 2697465 | 2698469 | - | 29 | 16 | CGGTTTATCCCCGCTGGCGCGGGGAACAC |
|  | Cas-Type IF | CRISPR-2 | 4036348 | 4039077 | - | 28 | 45 | TTTCTAAGCTGCCTACTCGGCAGTGAAC |
|  |  | CRISPR-3 | 4048055 | 4049407 | + | 28 | 22 | GTTCACTGCCGAGTAGGCAGCTTAGAAA |
|  |  | CRISPR-4 | 4054538 | 4055465 | - | 28 | 15 | TTTCTAAGCTGCCTACACGGCAGTGAAC |
|  | Cas- Type IIIA | CRISPR-5 | 2145323 | 2145993 | + | 37 | 9 | GTCCGTAAGGACGTTCCCTGACTGAAGGGATTAAGAC |
|  |  | CRISPR-6 | 2154634 | 2154938 | + | 23 | 4 | GGACTTGCCCTGACTGAAGGGAT |
|  | Orphan CRISPR | CRISPR-7 | 2442007 | 2443257 | - | 29 | 20 | CGGTTTATCCCCGCTGGCGCGGGGAACAC |
