# Supplementary material for: Genomic and Phenotypic Biology of Novel Strains of Dickeya zeae Isolated From Pineapple and Taro in Hawaii: Insights Into Genome Plasticity, Pathogenicity, and Virulence Determinants
Source: Front Plant Sci. 2021 Aug 11;12:663851. doi: 10.3389/fpls.2021.663851 (PMC8386352; doi:10.3389/fpls.2021.663851)
Supplement: Supplementary Table 4 — Identification of the secondary metabolite gene clusters using AntiSMASH in five genomes of Dickeya species. [file Table_4.DOCX]

| **Secondary metabolite gene clusters** | A5410 | PL65 | EC1 | Ech586 | MS2 |
| --- | --- | --- | --- | --- | --- |
| Ind-vfm-expI | + | + | + | + | + |
| Achromobactin | + | + | + | + | + |
| Chrysobactin | + | + | + | + | + |
| Cyanobactin | + | + | + | + | + |
| Betalactone | **+** | + | - | + | + |
| Bacteriocin (TfuA) | + | + | - | + | + |
| Arylpolyene | + | + | - | + | - |
| Bicornutin A1/A2 | - | - | + | - | - |
| Oocydin | - | - | + | - | - |
| Zeaemine | - | - | + | - | - |
| Luminmide | + | - | - | - | - |

**Table S4**. The secondary metabolite gene clusters identified with AntiSMASH in five genomes of *Dickeya* sp.
