# Supplementary material for: Genomic and Phenotypic Biology of Novel Strains of Dickeya zeae Isolated From Pineapple and Taro in Hawaii: Insights Into Genome Plasticity, Pathogenicity, and Virulence Determinants
Source: Front Plant Sci. 2021 Aug 11;12:663851. doi: 10.3389/fpls.2021.663851 (PMC8386352; doi:10.3389/fpls.2021.663851)
Supplement: Supplementary Table 5 — Prophages identified in D. zeae strains using Phaster. [file Table_5.DOCX]

**Table S5.** Prophages identified in *Dickeya zeae* strains using Phaster.

|  | **Region** | **Length** | **Completeness** | **Score** | **Specific Keyword** | **Position** | **Total protein** | **Phage Hit Proteins** | **Hypothetical Proteins** | **Bacterial Proteins** |
| --- | --- | --- | --- | --- | --- | --- | --- | --- | --- | --- |
| EC1 | 1 | 5.3 Kb | incomplete | 20 | transposase | 115517-120824 | 7 | 6 | 1 | 0 |
|  | 2 | 51.4 Kb | questionable | 90 | tail | 2287507-2338943 | 50 | 43 | 4 | 3 |
|  | 3 | 26.3 Kb | intact | 150 | tail, plate, head | 2446171-2472491 | 31 | 25 | 2 | 4 |
|  | 4 | 41 Kb | intact | 96 | integrase, lysin | 2780889-2821964 | 54 | 50 | 2 | 2 |
|  | 5 | 34.8 Kb | incomplete | 30 | integrase | 2815594-2850463 | 19 | 11 | 4 | 4 |
| MS2 | 1 | 11.1 Kb | incomplete | 50 | transposase, integrase, tail | 870870-882067 | 12 | 10 | 1 | 1 |
|  | 2 | 31.8 Kb | incomplete | 30 | integrase | 1667384-1699190 | 19 | 10 | 4 | 5 |
|  | 3 | 25.8 Kb | intact | 150 | tail, plate, transposase, head | 2665411-2691264 | 32 | 25 | 3 | 4 |
| Ech586 | 1 | 30.7 Kb | intact | 130 | integrase, portal, terminase, capsid, tail | 818289-849052 | 41 | 33 | 8 | 0 |
|  | 2 | 26.3 Kb | intact | 150 | tail, plate | 2010814-2037121 | 33 | 24 | 6 | 3 |
|  | 3 | 12.6 Kb | incomplete | 40 | integrase, head, tail | 2701746-2714372 | 11 | 7 | 4 | 0 |
|  | 4 | 19.1 Kb | incomplete | 40 | tail, integrase | 4293935-4313071 | 12 | 9 | 1 | 2 |
| A5410 | 1 | 32.4 Kb | intact | 96 | tail | 716647-749116 | 46 | 41 | 4 | 1 |
|  | 2 | 56.2 Kb | intact | 107 | lysin, tail | 2103140-2159365 | 53 | 48 | 3 | 2 |
|  | 3 | 24.1 Kb | incomplete | 20 | integrase | 2578494-2602689 | 15 | 8 | 3 | 4 |
| PL65 | 1 | 46.9 Kb | incomplete | 50 | integrase, transposase | 111294-158210 | 25 | 12 | 6 | 7 |
|  | 2 | 12 Kb | questionable | 90 | tail, coat | 785540-797603 | 15 | 9 | 6 | 0 |
|  | 3 | 4,9 Kb | questionable | 75 | coat | 797584-802575 | 6 | 0 | 0 | 0 |
|  | 4 | 45.5 Kb | intact | 100 | lysin, tail | 1033741-1079338 | 52 | 47 | 2 | 3 |
|  | 5 | 46.9 Kb | questionable | 80 | tail, lysin, integrase | 2287028-2333972 | 48 | 42 | 5 | 1 |
|  | 6 | 8.4 Kb | incomplete | 60 | transposase, head, tail | 2578494-2602689 | 13 | 8 | 0 | 5 |
